# Dietary iron and metal-based growth differentially modulate growth and gut microbiome of weaned piglets

**DOI:** 10.1186/s42523-026-00561-w

**Published:** 2026-04-11

**Authors:** Shya Navazesh, Anneliek ter Horst, Weizhang Wen, C. Titus Brown, Peng Ji

**Affiliations:** 1 Department of Nutrition, University of California Davis, Davis, CA USA; 2 Department of Population Health and Reproduction, University of California Davis, Davis, CA USA

**Keywords:** Zinc oxide, Growth promoter, Iron supplementation, Postweaning pig, Gut microbiome, Postweaning diarrhea

## Abstract

**Background:**

Weaning-associated gut dysbiosis significantly contributes to increased susceptibility to enteric infections in postweaning pigs. While Fe, Zn and Cu are essential micronutrients for bacteria including pathogens, the exact effects of transition metal restriction and excessive exposure on gut dysbiosis and pathogen virulence during weaning transition remain unclear. This study investigated how dietary iron and pharmacological levels of zinc and copper affect dynamic changes of gut microbiota in postweaning piglets experimentally challenged with enteric pathogen.

**Results:**

Fifty weanling pigs were stratified and randomized to five dietary treatments for 24 days (d). The experimental diets included a control diet (Con) containing 25, 139, and 141 mg/kg of Cu, Fe, and Zn, respectively, a low-iron diet (19 mg Fe/kg, LFe), a high-iron diet (1219 mg Fe/kg, HFe), a high-copper diet (257 mg Cu/kg, HCu), and a high-zinc diet (2631 mg Zn/kg with 2490 mg/kg from ZnO, HZn). The Con diet meets all nutrient requirements of nursery pigs, and the other diets were formulated based on the Con diet by removing or supplementing respective metals in mineral premix. All pigs were orally administered with enterotoxigenic *E. coli* (ETEC) once daily on d13–d16. Fecal microbiome was analyzed through 16 S rRNA sequencing on d1, 6, 12, 15, 18 and 24. Although the overall clinical signs of ETEC infection was moderate, the HFe and HCu pigs had lower diarrheal frequency than the Con pigs (*P* < 0.05). Fecal shedding of pathogen did not differ across treatments. Fecal microbiome showed the least changes in Shannon diversity in LFe pigs compared to the HFe and HCu groups over time (*P* < 0.05), while the HZn group showed the lowest Shannon diversity relative to groups Con, HFe, and HCu (*P* < 0.05). The β-diversity differed between LFe and HFe groups only at d24, whereas HZn reduced both α- and β-diversity beginning at d12 and d6, respectively (*P* < 0.05). Differential abundance analysis revealed greater abundance of *Campilobacteria* and *Escherichia-Shigella* in LFe pigs compared with the HFe group (*P* < 0.05), while HZn treatment was associated with *Bacteroidota* dominance and broad reductions in many taxa (*P* < 0.05).

**Conclusions:**

The pharmacological level of ZnO has profound effects on gut microbiome characterized by reduced diversity and *Bacteroidota* dominance. Despite moderate clinical signs, these results highlight the role of dietary iron and metal-based growth promoters in shaping gut microbiota and modulating resilience to infection.

**Supplementary Information:**

The online version contains supplementary material available at 10.1186/s42523-026-00561-w.

## Background

In swine production, the abrupt weaning to a solid diet primarily based on plant ingredients causes profound disruption of milk-oriented gut microbiota. Weaning-associated gut dysbiosis diminishes colonization resistance against pathogens and thereby increases susceptibility to infections. Enterotoxigenic *E. coli* (ETEC) is the leading cause for postweaning diarrhea (PWD), and explains the largest treatment antibiotic use in swine production [1, 2]. Since the ban on antibiotic growth promoters, pharmacological level of ZnO and, to a lesser extent, copper sulfate have been increasingly used in nursery diets to promote growth and prevent PWD in swine production [3, 4]. Despite their efficacy, the underlying mechanisms remain incompletely understood [5]. The beneficial effects of metal-based growth promoters have been ascribed to their role in modulating host immunity, interactions with gut bacteria and potential anti-microbial properties [4, 5], the exact impacts of excessive dietary exposure to transition metals on taxonomic composition of gut microbiota have not been fully characterized. High dose of heavy metals is toxic to bacteria and thus exerts persistent selective pressure for adaptation. Besides concerns over environmental accumulation, excessive ZnO has been linked to co-selection of heavy metal and antimicrobial resistance (AMR) in environmental and gut bacteria [6–8].

Iron is an essential nutrient for both pigs and most gut microbes including pathogens. Although nursery pigs (7–11 and 11–25 kg) requires 100 mg Fe/kg to meet growth requirement, a typical nursery diet usually contains two to five folds of recommended iron levels [9, 10]. Excess iron supply in postweaning diets likely reflects both the high intrinsic iron content of certain feed ingredients (e.g., limestone, dicalcium phosphate) and intended additional supplements due to perceived low bioavailability of plant-derived iron. Because suckling pigs receive iron supplementation parenterally and sow milk provides minimal iron [10], intestinal iron availability may substantially increases after consumption of iron-rich postweaning diet. As an essential nutrient for most microbes, altering iron availability has been shown to modulate gut microbiota and virulence of bacterial pathogens [11, 12]. Iron deprivation has been shown to profoundly reduce microbial diversity, with certain bacterial families, such as *Prevotellaceae* and *Porphyromonadaceae*, particularly sensitive to iron deprivation [13]. Conversely, iron supplementation in rodents led to significant reduction in relative abundance of *Lactobacillus* [14]. In human infants from regions with high infection prevalence, iron fortification has been associated with increased fecal pathogen load and elevated calprotectin levels, indicating greater intestinal inflammation [15]. Studies in rodent and in vitro models demonstrate that iron availability modulates *E. coli* pathogenicity by altering the expression of adhesion factors and toxins [16, 17], whereas iron deprivation stimulates fimbrial colonization factors and hemolysin in *E. coli* pathogens in vitro [18, 19]. Interestingly, supranutritional level of dietary iron supplementation completely protected mice from greater than lethal dose of *Citrobacter* by attenuating pathogen virulence, inducing asymptomatic infection [20]. However, limited research has investigated how altering dietary iron affects gut dysbiosis and infection risk in piglets during weaning transition.

This study aims to investigate the effects of dietary iron and pharmacological levels of metal-based growth promoters on growth performance, susceptibility to infection, and dynamic changes in gut microbiome in early postweaning pigs challenged ETEC pathogen.

## Methods

### Experimental design

All animal procedures were reviewed and approved by the Institutional Animal Care and Use Committee (IACUC #22720) at the University of California, Davis (UC Davis). A total of 50 weanling pigs (18 gilts and 32 barrows; 21–24 days (d) of age; body weight = 6.62 ± 1.07 kg) were selected from the Swine Teaching and Research Center at UC Davis and used in the study. All pigs were confirmed for genetic susceptibility to F18 ETEC through genotyping *FUT1* polymorphism using the method described in Kruezer et al. (2013) [21]. On the day of weaning, pigs were transferred to the Cole large animal facility at UC Davis and were housed in individual pens (0.61 m × 1.22 m) for 24 d including 12 d before and 12 d after the first ETEC inoculation. Pigs had free access to feed and water during the study. Animal rooms were equipped with fans and heaters to achieve the desired temperature for nursery pigs. A 12-h light cycle starting at 0700 h was automatically controlled in all animal rooms.

Piglets were stratified by sex and weaning body weight and randomly assigned to one of five dietary treatments (*n* = 10/treatments). Experimental diets were control diet (Con) that contains 25, 139, and 141 mg/kg of Cu, Fe, and Zn, respectively, low-iron diet (LFe, 19 mg Fe/kg), high-iron diet (HFe, 1219 mg Fe/kg), high-copper diet (HCu, 257 mg Cu/kg), and high-zinc diet (HZn, 2631 mg Zn/kg including 2490 mg Zn/kg from ZnO). The LFe, HFe, HCu, and HZn diets shared the same ingredient composition as the Con diet except that the respective metals were either removed from (e.g., LFe) or added in extra in the mineral-vitamin premix to achieve the desired levels. For HZn diet, the high zinc level was achieved by supplementing ZnO. The Con diet consisted of corn (~ 57%), non-fat dry milk (30%), dried whey (5.4%), and soy protein concentrate (4%) as the main ingredients. Milk product ingredients were higher than the levels commonly used in nursery diets. This was intended to reduce iron level in the basal diet, so that formulating LFe can be achieved by simply removing iron supplement from premix without altering main ingredients. The Con diet meets all nutrient requirements of nursery pigs. Ingredient composition and mineral (Fe, Cu, and Zn) concentrations of experimental diets were presented in Table S1. To prevent iron deficiency anemia which compromises host defense to infections independent of gut microbial modulation, LFe pigs were intramuscularly injected with iron dextran (100 mg Fe/1-mL injection) at d 2 and 7 during the study. Therefore, LFe treatment was the combined use of a low-iron diet and parenteral iron injection to evaluate how restricting intestinal iron availability affects gut microbiota and colonization resistance to ETEC infections. Starting from d 13, all pigs were orally administered an F18 ETEC inoculant (10^10^ colony-forming unit (CFU)/3-mL) once daily for 4 consecutive days. The F18 ETEC was originally isolated from a field disease outbreak by the University of Montreal (isolate number: ECL22131). The pathogenic strain express heat-stable enterotoxins (EAST-1, STb1) and heat-labile enterotoxin (LTlh-4).

### Sample and data collection

Body weight was measured at baseline (d 1), d 6 and every 3 days thereafter. Alertness and fecal consistency were scored twice daily throughout the study by the same researcher who was well-trained and had prior experience with both scoring systems. Alertness was scored following a 3-point scale (1 = normal and alert, 2 = slightly depressed, lethargic with reduced appetite, and 3 = severely depressed and recumbent). Fecal consistency was scored based on a 5-point scale (1 = normal feces, firm and well-formed, 2 = moist feces and slightly soft, 3 = mild diarrhea with soft and pasty feces, 4 = severe diarrhea with liquid feces containing some solid content, 5 = watery diarrhea that is completely liquid with undigested feed particles). Feed intake was recorded for each pig for the periods of d 1–7, 8–11, 12–17, and 18–23. Average daily feed intake (ADFI) was calculated for each period and average daily gain (ADG) was calculated as the changes in body weight divided by the number of days within each period. The feed conversion ratio (FCR) was calculated by dividing the ADFI by the ADG.

Fecal samples were collected through rectal swabbing on d 1, 6, 12, 15, 18, 21, and 24 and stored at -80˚C until analysis. Blood samples were collected from jugular vein using heparinized vacutainers (BD, Franklin Lackes, NJ) on the same days of fecal sampling and tested for hemoglobin (Hb) and hematocrit (Hct). On d 24, piglets were anesthetized after an intramuscular injection of Telazol/ketamine/xylazine mixture (2:1:1 ratio) at 0.05 mL/kg body weight. Pigs were subsequently euthanized with an intracardiac injection of sodium pentobarbital solution (Fatal-plus, ≥ 100 mg/kg body weight). Tissue samples were collected and stored at -80˚C prior to analysis.

### Hematological indices

Hemoglobin concentration was analyzed using Drabkin’s reagent (Sigma-Aldrich, St. Louis, MO) following the manufacturer’s instructions with modifications on reagent volumes for a microplate application (250 µL). Absorbance was detected at 540 nm using a microplate reader (Synergy HTX, BioTek, Winooski, VT). Hemoglobin concentrations were calculated based on absorbance of a serially diluted porcine hemoglobin standard samples (Sigma-Aldrich, St. Louis, MO). Hematocrit was determined using a micro-Hct centrifuge.

### Analysis of tissue and fecal mineral concentrations

Liver, spleen, heart, and fecal samples (50–200 mg) were digested in 16 N nitric acid (Trace mineral grade, Thermo Fischer Scientific, Waltham, MA) for 72 h. Samples were heated to 180˚C until complete evaporation of acid, and subsequently reconstituted into 5 mL with Milli-Q water. Reconstituted samples were analyzed for Cu, Fe, Mn, and Zn via an inductively coupled plasma optical emission spectrometer (ICP-OES, iCAP 6000, ThermoScientific). The analysis was calibrated at the beginning and then every 20 samples with blank solution. Mineral concentrations were calculated based on a standard curve and normalized with tissue weight.

### Fecal culture and enumeration of E. coli and β-hemolytic coliforms

MacConkey agar and Sheep blood agar were used for verification and enumeration of lactose-fermenting (*E. coli*) and β-hemolytic bacteria, respectively, from fecal cultures. Fecal swabs collected on d 12 (pre-inoculation), 15, 18, 21, and 24 were streaked on agar plates following a quadrant streak method. Plates were incubated at 37˚C for 24 h prior to evaluation. Plates were visually scored from 0 to 8, with 0 indicating no bacterial growth and 8 indicating heavy bacterial growth in all quadrants. The percentage of β-hemolytic coliforms was calculated as the ratio between the growth scores of β-hemolytic coliforms and lactose-fermenting coliforms.

### Fecal DNA extraction

The DNA was extracted from fecal samples using the QIAamp PowerFecal Pro DNA Kit (Qiagen, Hilden, Germany). Approximately 250 mg of fecal samples were homogenized using a TissueLyser II (Qiagen, Hilden, Germany) at 25 Hz for 10 min. Subsequent steps were performed according to the manufacturer’s instructions. The ZymoBIOMICS Microbial Community Standard was included as a positive control and extracted alongside fecal samples. DNA quality was assessed on a 1% agarose gel containing SYBR ™ Safe (Thermo Fisher Scientific, Waltham, MA), and the concentration and purity were quantified using a NanoDrop spectrophotometer.

### PCR amplification and 16SrRNA sequencing of fecal DNA

Fecal DNA samples were amplified for V4 regions of bacterial 16 S rRNA genes through PCR using the primers 515 F (5′-XXXXXXXXGTGTGCCAGCMGCCGCGGTAA-3′), which included an 8-nt poly-X sequence as a unique barcode for each sample followed by a 2-nt Illumina adapter (bold), and 806R (5′-GGACTACHVGGGTWTCTAAT-3′). The 25-µL PCR reaction contained 2 µL DNA template, 9.5 µL nuclease-free water, 12.5 µL GoTaq G2 Green Master Mix (Promega, Madison, WI), and 0.5 µL of each primer (10 µM) and was performed in triplicates. Nuclease free water was used as negative control. Following amplification, samples were loaded into a 2% agarose gel electrophoresis to verify amplicon size and evaluate band intensity. Library was prepared by pooling all fecal DNA samples, subsequently purified through the QIAquick PCR Purification Kit (Qiagen, Hilden, Germany), and then submitted for 16 S rRNA sequencing at the DNA Technologies and Expression Analysis Core Laboratory at UC Davis. Sequencing was performed using the Illumina MiSeq 500 platform (Illumina, Inc., San Diego, CA) with paired-end 250 bp reads. A total of 12.6 million reads passing initial filtering with an overall Q30 > 90.6%. The 16 S rRNA sequencing data have been deposited in the NCBI Sequence Read Archive (SRA) database under BioProject ID PRJNA1330844. Project information is accessible in the following link: https://www.ncbi.nlm.nih.gov/sra/PRJNA1330844.

### Microbiome data analysis

Raw DNA sequences were analyzed using Quantitative Insights into Microbial Ecology 2 (QIIME2, 2023.5) [22]. Data were demultiplexed using the cutadapt demux-paired plugin, revealing a total of 11,861,773 reads and a mean of 7,832 reads per sample. Then, data were subsequently denoised with the DADA2 plugin (q2-dada2) to identify unique 16 S rRNA gene amplicon sequence variants (ASV) [23]. To optimize read quality, forward and reverse sequences were trimmed to 200 bp and 180 bp, respectively. Taxonomy was classified using the classify-sklearn plugin using a pre-trained Naïve Bayes classifier against the Silva (v.138) reference database with 99% OTU clustering [24–26]. Reads mapped to nontarget organisms, such as mitochondria and chloroplasts, and ASV present in only 1 sample were then removed, resulting in the identification of a total of 1398 ASV. Alpha rarefaction plots were created using the alpha-rarefaction plugin to determine sampling depth for diversity analyses. Phylogenetic trees were constructed using the align-to-tree-mafft-fasttree plugin, where representative ASV’s were aligned using MAFFT and an unrooted tree was constructed using masked alignments in fasttree2 [27, 28]. Alpha-diversity (Shannon’s H Index and Faith’s Phylogenetic Diversity Index) and beta diversity metrics (Jaccard distance, Bray-Curtis dissimilarity, unweighted UniFrac and weighted UniFrac) were estimated using q2-diversity after samples were rarefied to 1600 sequences per sample [29, 30].

### Statistical analyses

Data of growth performance, hematological indices, and tissue and fecal mineral concentrations were analyzed in GraphPad Prism (v.10.5.0). Data were assessed for equal variance and normal distribution using homoscedasticity and Q-Q plots. All data were fit for both assumptions and were subsequently analyzed using a mixed effects model. The model included the fixed effects of treatment and day and the treatment by day interactions with subject as a random term. Repeated measures were incorporated into the model for variable measured over time and Tukey’s test was used to adjust for multiple comparisons. The frequency of diarrhea was calculated as the total days of diarrhea observed within each treatment during the study. Diarrhea was represented as a fecal score ≥ 3. Pairwise comparisons were analyzed using Fisher’s exact test. Statistical significance was declared at p or adjusted-*p* ≤ 0.05.

Differences in alpha and beta diversity of fecal microbiome were detected using the Kruskal–Wallis test and pairwise Permutational Multivariate Analysis of Variance (PERMANOVA), respectively. P values were adjusted for multiple comparisons using the Benjamini-Hochberg false discovery rate (BH-FDR) and statistical significance was determined at *p* < 0.05. The q2-longitudinal plugin in QIIME2 was utilized to perform longitudinal analysis of alpha diversity (Shannon index) and beta diversity (Bray-Curtis and Weighted UniFrac distances). Temporal changes in diversity metrics within each treatment were evaluated using two approaches: (1) Metric differences were calculated between successive timepoints. (2) Metric differences were calculated between the baseline (d 1) and each of the subsequent time points. Calculated differences from the two approaches were evaluated using a linear mixed model with the Con group as a reference. To determine treatment effects within paired timepoints, within-subject pairwise comparisons were analyzed using a Wilcoxon signed-rank test. Then, to evaluate whether the temporal changes differed across treatment groups, a Mann-Whitney U test was performed. P-values were adjusted using BH-FDR correction [31]. Relative abundances were calculated using the mean ceiling of samples within a treatment group. Differential abundance of raw taxonomic counts was assessed using DESeq2 (v.1.46.0). Pairwise comparisons were extracted and plotted as log2 fold change, and p-values were adjusted using Benjamini-Hochberg. Results were plotted as log2 fold change using ggplot2 (v.3.5.2) in R (v.4.4.3).

## Results

### Growth performance and hematological changes

There were no significant differences in alertness scores across treatments during the study (Figure S1A). The growth performance did not differ among treatments (Figures S1B-E). On d12, the LFe and the HFe groups had higher Hb compared to the Con, HCu, and HZn groups (Figure S1F, *p* < 0.05). Hematocrit was also significantly lower in the HCu group compared to the LFe and the HFe groups (Figure S1G, *p* < 0.01). On d18, HCu group had lower Hb and Hct compared to the HFe group and lower Hb compared to the HZn group, but no differences were detected among other groups. By d24, LFe pigs had lower Hb and Hct levels compared to HFe and HZn groups. The HCu pigs had the lowest Hb and Hct among all treatment groups (*P* < 0.05).

### Frequency of diarrhea and fecal pathogen abundance

**Fig. 1:**
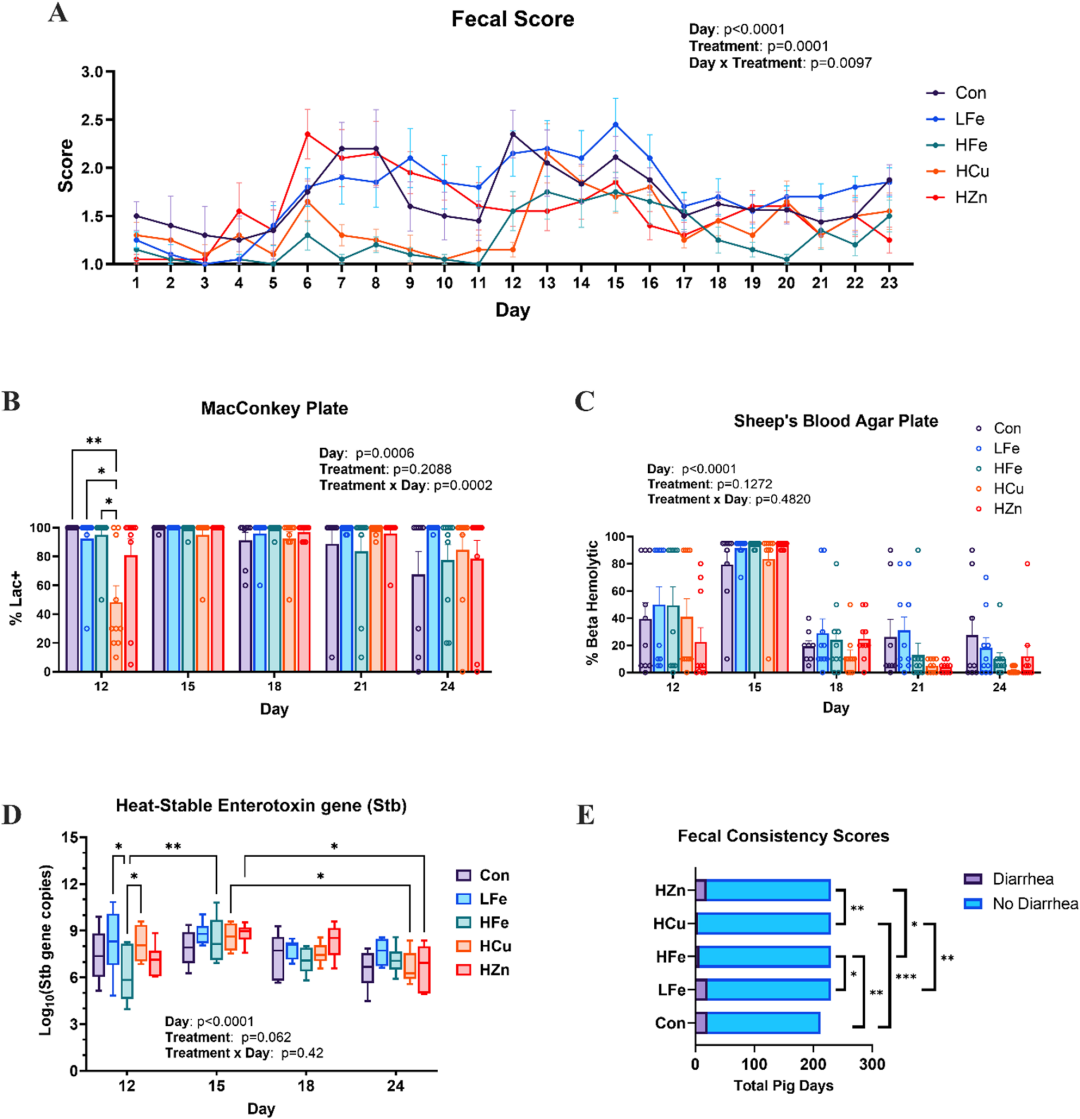
Effects of dietary treatment and ETEC challenge on fecal consistency and fecal shedding of pathogen. **(A)** Changes in average daily fecal score (5-point scale) throughout the study, **(B)** Fecal swab plating on MacConkey agar, **(C)** Fecal culture for lactose fermenting and hemolytic bacteria using MacConkey agar and sheep blood agar, respectively **(D)** Log10 transformed copy numbers of the Heat-stable enterotoxin (Stb) gene *est-II* in fecal DNA (whiskers: 5th – 95th percentiles, box edges: 25th – 75th percentiles, line in box: median) **(E)** Total pig days of diarrhea (fecal score ≥ 3) for each treatment group. Pairwise comparison: *, *p* < 0.05; **, *p* < 0.01; ***, *p* < 0.001; ****, *p* < 0.0001. Con, Control diet; LFe, Low Iron Diet; HFe, High Iron diet; HCu, High Copper diet; HZn, High Zinc diet

Significant main and interaction effects were observed on fecal scores (Fig. 1A). An increase in fecal score was observed in the Con, LFe, and HZn groups as early as d6, whereas increases in the HFe and HCu groups did not appear until d12. The HFe pigs had lower fecal scores than the HZn or LFe groups on various days (*P* < 0.05). Additionally, the HCu group had lower scores on d12 compared with the Con and LFe groups (*P* < 0.05). Despite changes in fecal consistency, the overall diarrheal illness was moderate in this study regardless of the treatment. A significant time effect was observed in fecal culture on both MacConkey and sheep blood agars, with d15 showing the highest colony levels, suggesting increased fecal shedding of lactose-fermenting and beta-hemolytic bacteria (Figs. 1B, C, *p* < 0.01). Heat-stable enterotoxin b, encoded by *est-II* gene, is a virulence factor of the F18 ETEC strain used in this study. Consistent with fecal culture results, qPCR analysis of fecal *est-II* abundance (log_10_-transformed) revealed a significant time effect, with d15 showing the highest abundance, and a trend toward a significant treatment effect (Fig. 1D). Diarrhea was defined as a fecal score ≥ 3. Both the HFe and HCu groups had significantly fewer total pig days of diarrhea compared with the other groups (Fig. 1E, *p* < 0.05).

### Tissue and fecal mineral concentrations

The HFe, HCu, and HZn treatments significantly increased hepatic concentrations of their respective metals compared with the other groups (Table S2, *p* < 0.01). Splenic Fe, Cu, and Mn, as well as heart Fe and Mn concentrations, were significantly affected by treatment (*P* < 0.05). The HCu group had the highest splenic Cu concentration but the lowest splenic iron concentration, which significantly differed from those in the HFe and HZn groups (*P* < 0.05). Similarly, the HCu group also had lower heart Fe concentration compared with the Con, HFe, and HZn groups (*P* < 0.05). Additionally, heart Mn concentration was lower in the HFe than in the LFe and HCu groups (*P* < 0.05).

Fecal Fe, Cu and Zn concentrations were significantly higher in the HFe, HCu, and HZn groups, respectively, than in the other groups across all sampling days (Table 1, *P* < 0.05). As expected, the LFe group had the lowest fecal Fe concentrations throughout the study. Interestingly, the HZn group consistently showed the lowest fecal Cu concentrations, despite varying levels of statistical significance across sampling days.

**Table 1:** Mineral concentrations (µmol/g) in fecal samples on d12, 18, 21, and 24

Mineral	Day	Con	LFe	HFe	HCu	HZn
Iron	12	6.5 ± 2.8^b^	2.2 ± 1^c^	40.2 ± 15.9^a^	7.1 ± 3.4^b^	3.9 ± 1.6^bc^
	18	4.8 ± 2.3^bc^	2.2 ± 0.6^c^	34.6 ± 17.2^a^	4.6 ± 2.2^b^	4.3 ± 2.0^bc^
	21	5.0 ± 3.4^b^	2.6 ± 1.3^b^	45.3 ± 20.8^a^	6.7 ± 5.0^b^	3.1 ± 1.8^b^
	24	6.8 ± 1.3^b^	3.5 ± 1.2^c^	47.3 ± 15.8^a^	8.0 ± 2.7^b^	5.3 ± 2.2^bc^
Copper	12	0.9 ± 0.3^b^	0.7 ± 0.4^b^	1.0 ± 0.2^b^	9.5 ± 1.8^a^	0.7 ± 0.3^b^
	18	0.9 ± 0.2^b^	0.9 ± 0.2^b^	1.0 ± 0.6^bc^	9.1 ± 2.1^a^	0.6 ± 0.1^c^
	21	1.0 ± 0.2^b^	1.0 ± 0.2^b^	1.1 ± 0.2^b^	10.3 ± 3.2^a^	0.6 ± 0.2^c^
	24	0.9 ± 0.2^b^	0.9 ± 0.2^b^	1.0 ± 0.2^b^	9.0 ± 2.1^a^	0.6 ± 0.3^b^
Zinc	12	5.9 ± 2.5^b^	4.9 ± 2.6^b^	6.6 ± 1.2^b^	6.5 ± 1.6^b^	73.0 ± 24.0^a^
	18	6.3 ± 0.9^b^	6.1 ± 1.3^b^	6.2 ± 1.4^b^	5.8 ± 0.5^b^	67.8 ± 13.0^a^
	21	7.1 ± 1.4^b^	6.4 ± 1.8^b^	6.5 ± 1.2^b^	6.6 ± 1.6^b^	65.6 ± 18.9^a^
	24	5.8 ± 1.1^b^	6.2 ± 1.1^b^	7.0 ± 1.8^b^	6.9 ± 1.9^b^	79.4 ± 37.1^a^
Manganese	12	2.1 ± 1.0^ab^	1.8 ± 0.9^ab^	2.6 ± 0.5^a^	2.4 ± 0.6^ab^	1.8 ± 0.6^b^
	18	2.2 ± 0.3^a^	2.2 ± 0.4^a^	2.3 ± 0.5^a^	2.2 ± 0.3^a^	1.6 ± 0.4^b^
	21	2.6 ± 0.4^a^	2.4 ± 0.5^a^	2.6 ± 0.5^a^	2.5 ± 0.7^a^	1.6 ± 0.5^b^
	24	2.0 ± 0.3	2.3 ± 0.4	2.4 ± 0.6	2.2 ± 0.6	1.6 ± 0.7

Values are presented as mean ± SD. Means within a row that do not share a superscript letter (a–c) are significantly different (*P* < 0.05)

Con, Control diet; LFe, Low Iron Diet; HFe, High Iron diet; HCu, High Copper diet; HZn, High Zinc diet

### Microbial diversity within timepoints

**Fig. 2:**
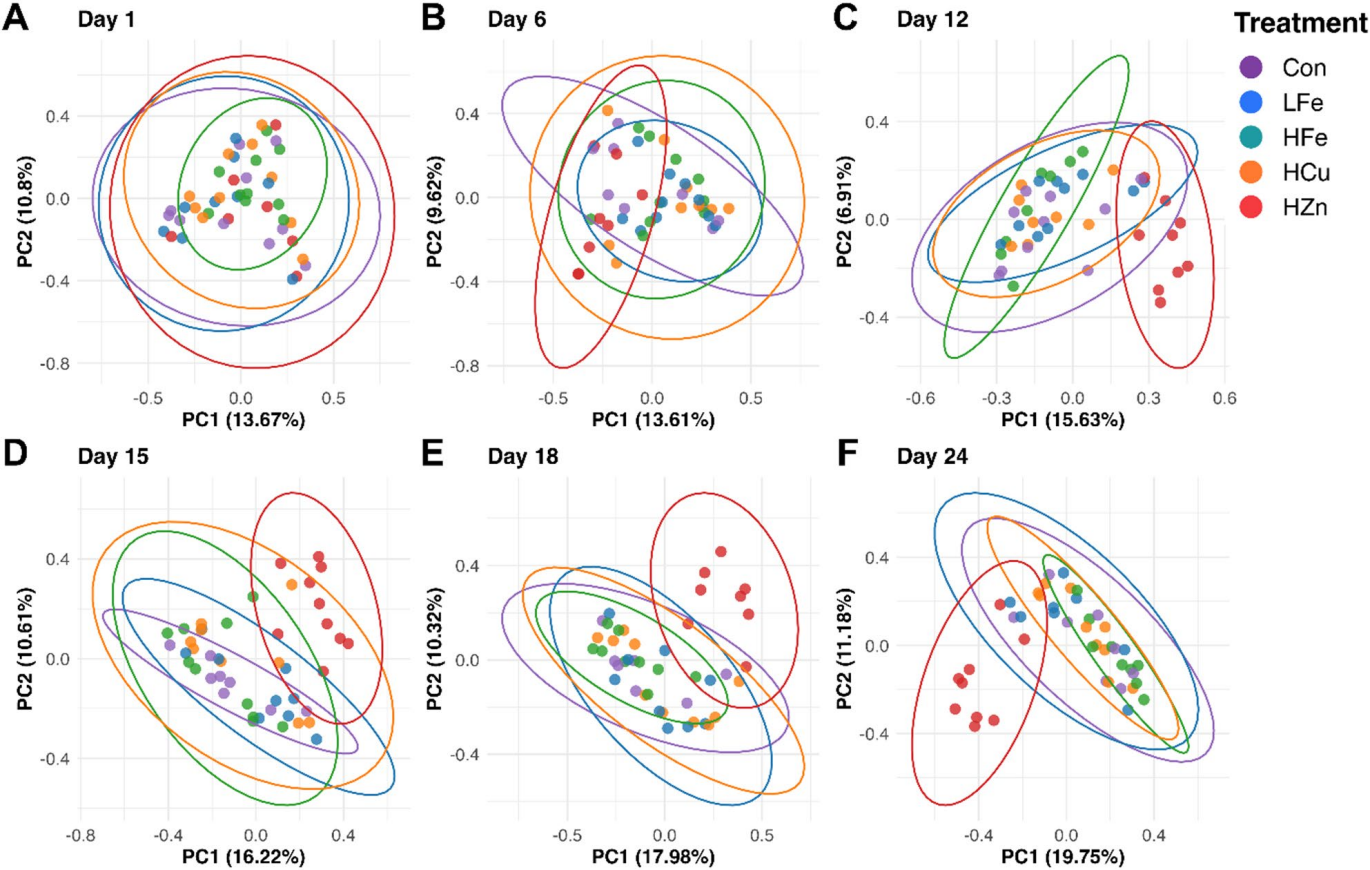
Effects of dietary treatment on β-diversity (Bray-Curtis dissimilarity distances) of fecal microbiome across study days. Data were shown in a principal coordinates analysis (PCoA) plot. The PCoA plots presented top two principal components and 95% confidence ellipses for samples from the same treatment. Con, Control diet; LFe, Low Iron Diet; HFe, High Iron diet; HCu, High Copper diet; HZn, High Zinc diet

**Fig. 3:**
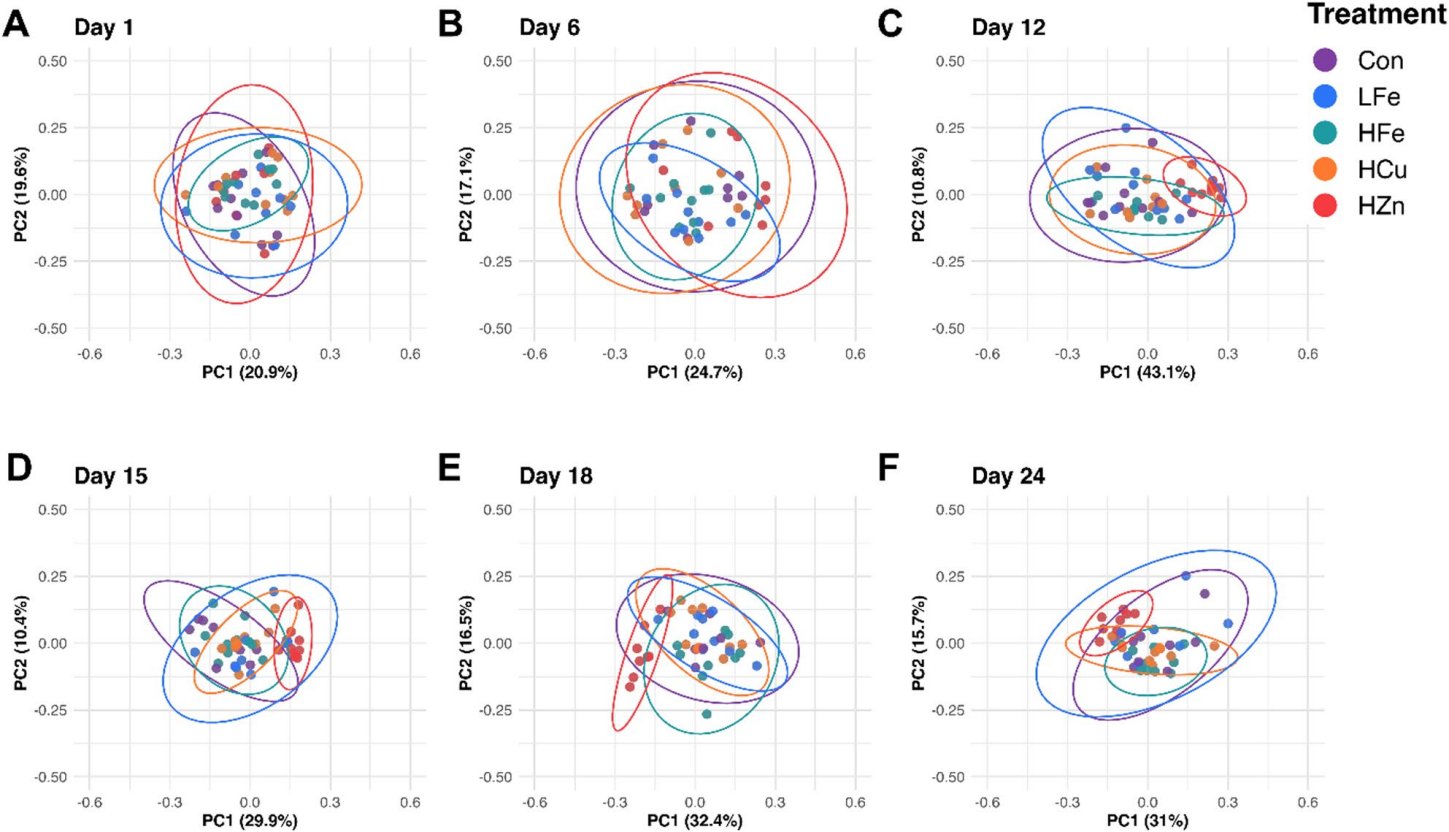
Effects of dietary treatment on β-diversity (Weighted UniFrac distances) of fecal microbiome across study days. Data were shown in a principal coordinates analysis (PCoA) plot. The PCoA plots presented top two principal components and 95% confidence ellipses for samples from the same treatment. Con, Control diet; LFe, Low Iron Diet; HFe, High Iron diet; HCu, High Copper diet; HZn, High Zinc diet

Beta diversity of the fecal microbiome was evaluated using Bray-Curtis dissimilarity and weighted UniFrac distances (Figs. 2 and 3). No differences in either distance metric were observed on d1. From d6 onward, both indices differed significantly between the HZn and the other groups (*P* < 0.01), with increased divergence sustained through the end of the study. On d24, the Bray-Curtis dissimilarity distance of the HFe group differed from those of the LFe and HCu groups (*P* ≤ 0.053).

Alpha diversity was assessed using Shannon’s Diversity Index and Faith’s Phylogenetic Diversity (PD) metrics (Table 2). Neither metric differed among treatments at baseline, suggesting similar fecal microbial diversity at weaning. At subsequent timepoints, the LFe group showed higher Shannon diversity than the other groups on d6 only, whereas the HZn group exhibited significantly lower alpha diversity in both metrics since d12 (q < 0.05).

**Table 2:** α-Diversity of fecal microbiomes within timepoints

Metric	Day	Con	LFe	HFe	HCu	HZn
Shannon	1	4.8 ± 0.8	5.2 ± 0.6	4.6 ± 0.7	4.8 ± 0.5	4.6 ± 0.6
	6	4.8 ± 0.3^a^	5.4 ± 0.3^b^	5.0 ± 0.3^ab^	4.8 ± 0.5^a^	4.2 ± 1.0^c^
	12	5.0 ± 0.7^ab^	4.9 ± 0.9^a^	5.5 ± 0.3^b^	5.2 ± 0.4^ab^	4.3 ± 0.3^c^
	15	5.1 ± 0.2^a^	4.9 ± 0.7^a^	5.0 ± 0.4^a^	5.1 ± 0.6^a^	4.2 ± 0.3^b^
	18	4.9 ± 0.3^a^	4.8 ± 0.4^a^	4.9 ± 0.5^a^	4.8 ± 0.4^a^	3.5 ± 0.7^b^
	24	4.8 ± 0.4^a^	4.7 ± 0.4^a^	5.1 ± 0.3^a^	4.9 ± 0.4^a^	3.5 ± 0.5^b^
Faith’s	1	7.8 ± 1.9	8.8 ± 1.9	8.0 ± 1.3	7.4 ± 1.2	7.2 ± 0.6
Phylogenetic	6	9.5 ± 1.1	10.3 ± 0.7	9.8 ± 1.4	9.8 ± 1.1	6.9 ± 4.1
Diversity	12	7.7 ± 1.4^a^	7.7 ± 1.5^a^	8.8 ± 1.2^a^	8.0 ± 0.8^a^	4.3 ± 0.4^b^
	15	8.7 ± 1.1^a^	7.9 ± 1.1^a^	8.5 ± 0.7^a^	8.0 ± 1.8^a^	4.9 ± 0.6^b^
	18	7 ± 1.0^a^	7.2 ± 0.5^a^	7.4 ± 0.9^a^	7.1 ± 1.1^a^	3.8 ± 0.6^b^
	24	6.7 ± 0.9^a^	6.4 ± 0.9^a^	7.4 ± 0.5^b^	6.9 ± 1.1^ab^	3.4 ± 0.6^c^

Values are presented as mean ± SD. Means within a row that do not share a superscript letter (a–c) are significantly different (*P* < 0.05)

Con, Control diet; LFe, Low Iron Diet; HFe, High Iron diet; HCu, High Copper diet; HZn, High Zinc diet

^a, b,c,^Within the Day, values that share no common letter differed significantly across treatments

### Temporal changes in fecal microbial diversity

**Fig. 4:**
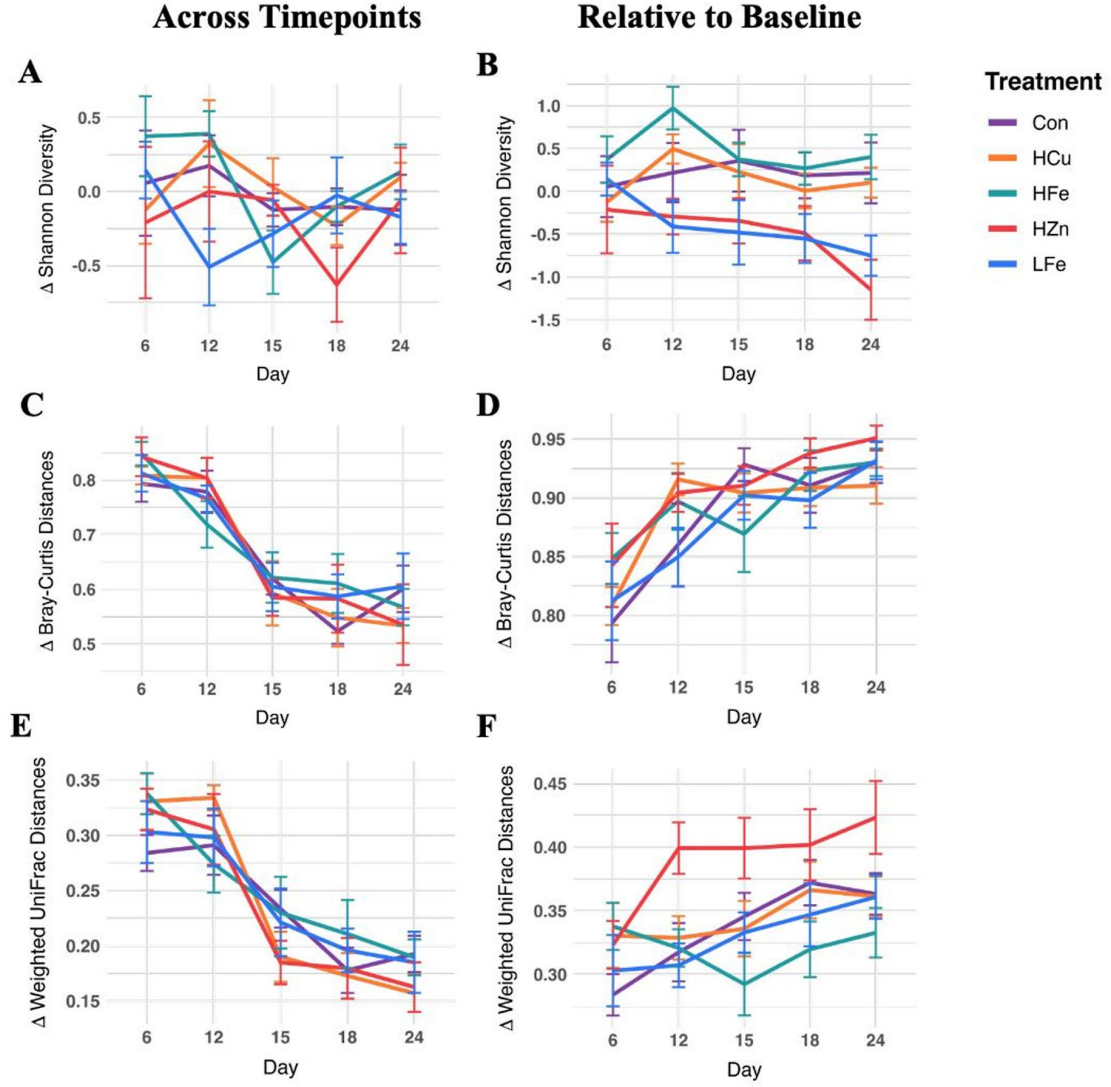
Effects of dietary treatment on successive or cumulative changes in fecal microbial diversity. Volatility plots showed changes in Shannon entropy **(A)**, Bray-Curtis Distances **(B)**, and Weighted UniFrac Distances **(C)** relative to the previous timepoint. Volatility plots showed changes in Shannon entropy **(D)**, Bray-Curtis Distances **(E)**, and Weighted UniFrac Distances **(F)** relative to the baseline (d1) value. Con, Control diet; LFe, Low Iron Diet; HFe, High Iron diet; HCu, High Copper diet; HZn, High Zinc diet

To evaluate microbial dynamics while accounting for baseline variation, we calculated the rate of change for Shannon diversity between successive time points (Fig. 4A). No significant main or interaction effects were observed across treatment groups. However, the HZn group exhibited a significant decline between d15 and d18, suggesting that while overall temporal variation was modest, short-term fluctuations were detected (Wilcoxon signed-rank test, W = 0, *p* < 0.05). We further performed pairwise comparisons between treatments. It revealed that from d6 to d12, the LFe group showed a significantly reduced change in Shannon diversity compared with the HFe group, indicating opposing diversity changes during this period (*P* < 0.05).

To evaluate cumulative treatment effects, changes in Shannon diversity were recalculated relative to baseline (d1) (Fig. 4B). Although no main effects were observed, significant treatment × day interactions were detected for the HZn and LFe groups (*P* < 0.05). The HZn group did not demonstrate within-group temporal shifts but showed reduced microbial expansion compared with other groups. From baseline to d12, the HZn group exhibited a significantly smaller change in Shannon diversity relative to the HFe group (*P* < 0.05). Similarly, from baseline to d24, the HZn group exhibited significantly lower changes compared with the Con, HFe, and HCu groups, indicating cumulative suppression of microbial diversity following ZnO supplementation (*P* < 0.05). The LFe group also trended toward reduced microbial expansion, demonstrating lower Shannon diversity at d24 relative to baseline (Wilcoxon signed-rank test, W = 0, *p* = 0.078) and significantly reduced changes compared with the HFe and HCu groups (*P* < 0.05). A similar trend was observed from baseline to d12 when compared with the HFe group (*P* = 0.055).

Temporal changes in beta diversity were next evaluated. Rates of change in Bray-Curtis dissimilarity between successive timepoints revealed a significant day effect but no treatment or interaction effects (Fig. 4C, *p* < 0.05). When distance metrics were recalculated relative to baseline to the baseline to evaluate cumulative effects (Fig. 4D), a significant day effect persisted (*P* < 0.05), whereas treatment and interaction effects remained nonsignificant. The HFe group trended toward a significant treatment × day interaction (*P* = 0.052), although pairwise comparisons did not reveal significant differences between treatments at any periods.

Analysis of weighted UniFrac distances across successive timepoints similarly revealed only a significant day effect (Fig. 4E, *p* < 0.05). When recalculated related to baseline, the HFe group showed significant treatment effect and treatment × day interaction (Fig. 4F, *p* < 0.05). Pairwise comparisons between treatments indicated that only the d1-12 period showed significantly greater change in phylogenetic distance in the HZn group compared with the LFe and HFe groups (*P* < 0.05), with trends toward significance compared with the HCu and Con groups (*P* = 0.10).

### Relative and differential abundance

**Fig. 5:**
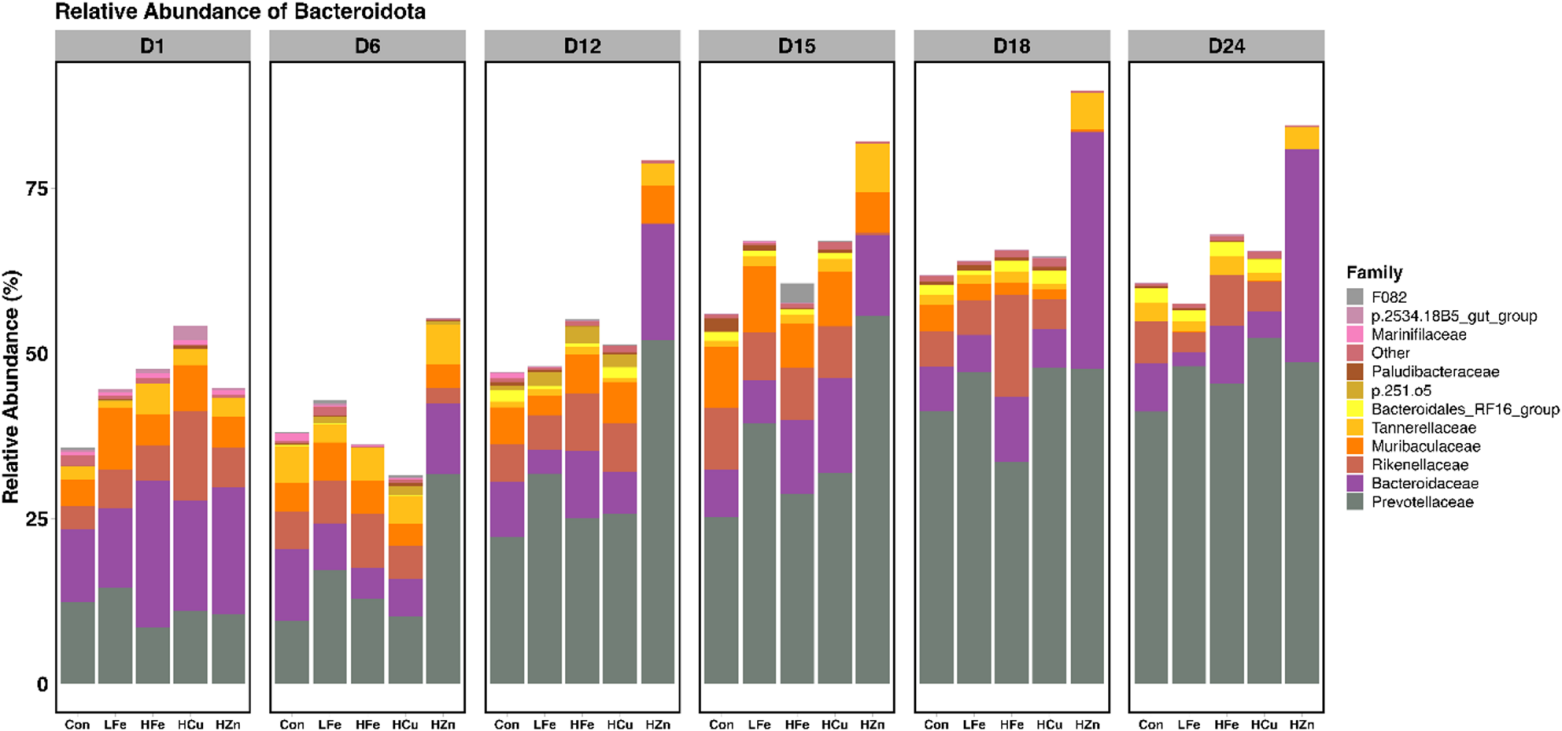
Average relative abundance of primary bacterial families of *Bacteroidota* across sampling days. Relative abundances were calculated using the mean ceiling of samples within a treatment group. Panels represent the day of sampling, d1, 6, 12, 15, 18, and 24. Con, Control diet; LFe, Low Iron Diet; HFe, High Iron diet; HCu, High Copper diet; HZn, High Zinc diet

**Fig. 6:**
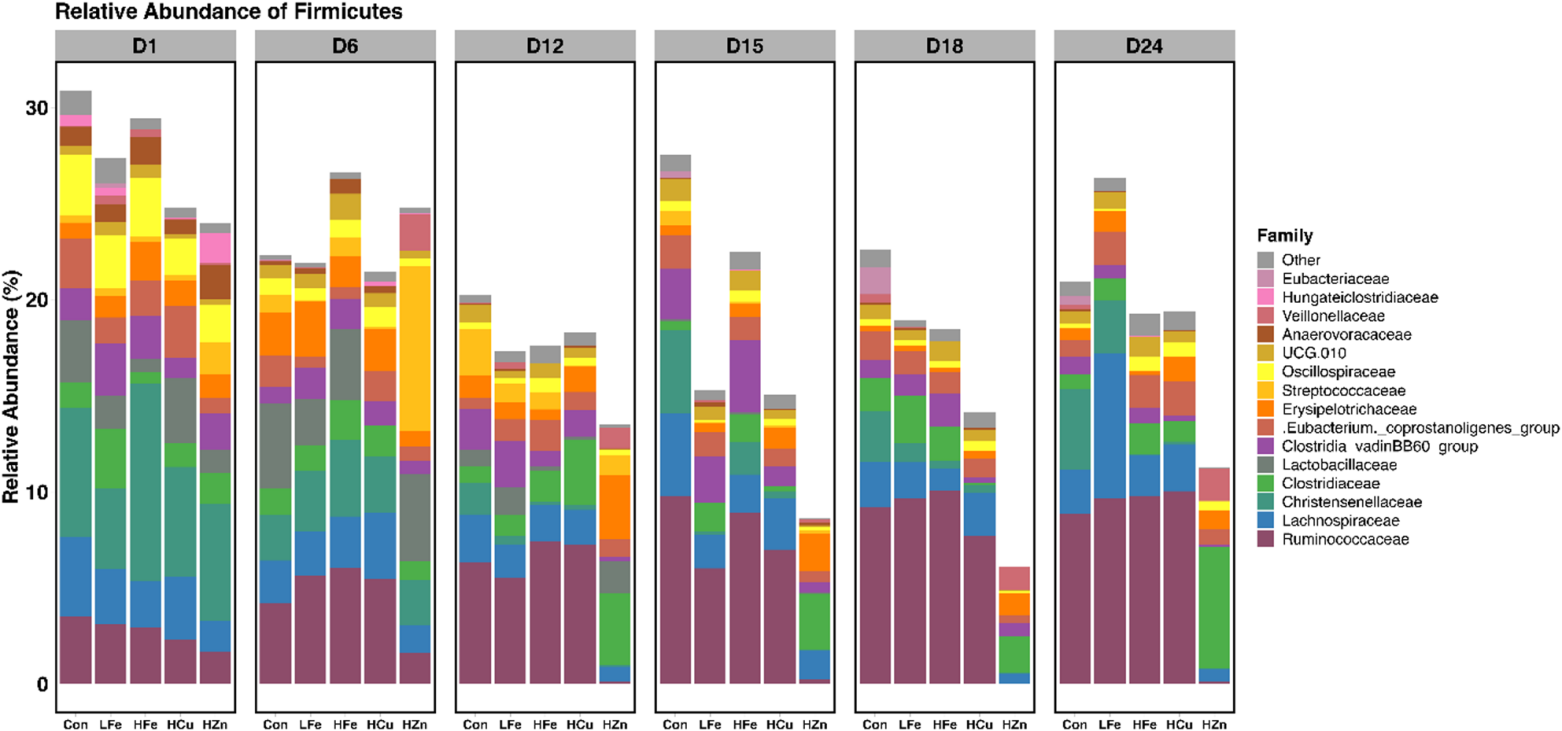
Average relative abundance of primary bacterial families of *Bacillota* across sampling days. Relative abundances were calculated using the mean ceiling of samples within a treatment group. Panels represent the day of sampling: d1, 6, 12, 15, 18, and 24. Con, Control diet; LFe, Low Iron Diet; HFe, High Iron diet; HCu, High Copper diet; HZn, High Zinc diet

*Bacillota* and *Bacteroidota* were the dominant phyla in the fecal microbiota of all treatments (Table S3). At the genus level, *Prevotella*, *Bacteroides*, and *Treponema* were the most abundant taxa throughout the study (Table S4). Differential abundance analysis revealed that the HZn group exhibited the most pronounced restructuring of microbial composition. Specifically, the HZn group was significantly enriched in *Bacteroidota* phylum on d6, 12, and 15 compared with the other groups (Fig. 5, *p* < 0.05), accompanied by concurrent reduction in *Verrucomicrobiota* (*P* < 0.05). Although *Bacillota* was slightly increased in the HZn group at d6 (24.8% vs. 22.3%, *p* < 0.05), it was significantly reduced by d18 relative to the control (6.1% vs. 22.6%, Fig. 6, *p* < 0.05).

**Fig. 7:**
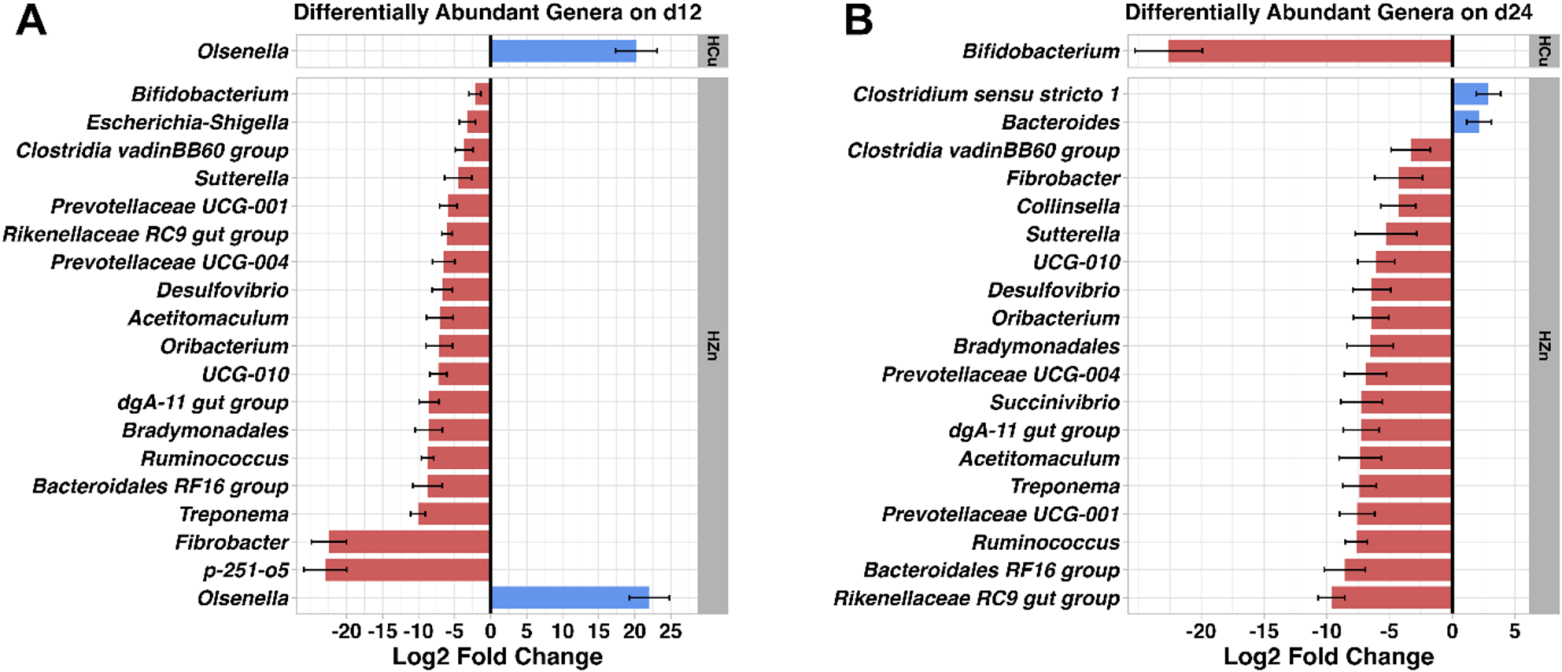
Differentially abundant genera on d12 **(A)** and d24 **(B)** across treatment groups represented as log2 fold change. Count data were analyzed using DESeq2 and only genera with a BH-FDR adjusted-*p* < 0.05 are shown. Con, Control diet; LFe, Low Iron Diet; HFe, High Iron diet; HCu, High Copper diet; HZn, High Zinc diet

These phylum-level shifts were reflected at the family level. On d6, *Prevotellaceae* (31.8% vs. 9.5%) and *Enterobacteriaceae* (8.6% vs. 1.3%) were significantly enriched in the HZn group relative to the Con group (*P* < 0.05). By d12, HZn treatment led to significant reductions in multiple families, including *Spirochaetaceae*, *Bradymonadales*, *Rikenellaceae*, *Ruminococcaceae*, *Bacteroidales RF16 group*, *UCG.010*, and *Desulfovibrionaceae* (*P* < 0.05), with corresponding genus-level reductions in *Ruminococcus*, *Treponema*, and *Rikenellaceae RC9 gut group* (Fig. 7A, *p* < 0.05). These reductions persisted across subsequent timepoints. On d15, *Atopobiaceae* (4.5% vs. 0.1%), *Tannerelaceae* (7.4% vs. 1.0%), *Prevotellaceae NK3B31* group (16.0% vs. 3.6%) and *Parabacteroides* (7.4% vs. 1.0%) were enriched in the HZn group relative to the Con group (*P* < 0.05). Additionally, *Olsenella* was significantly higher in the HZn group compared with the Con, HFe and HCu groups; this family was undetectable in the later two groups (*P* < 0.05). On d18, although many taxa remained suppressed in the HZn group, *Bacteroides* abundance increased markedly (35.9% vs. 6.7%, *p* < 0.05), followed by increased *Bacteroidaceae* at d24 (32.2 vs. 7.4%, *p* < 0.05). On d24, the HZn group showed increased abundance of *Clostridium sensu stricto 1* (6.3% vs. 0.8%) and *Dialister* (1.6% vs. 0.2, Fig. 7B, *p* < 0.05). Together, these results indicate that pharmacological level of ZnO induced sustained gut microbial restructuring characterized by reduced diversity, dominance of *Bacteroidota*, and a temporal shift from *Prevotellaceae* towards *Bacteroidaceae*.

In contrast, relatively few differentially abundant taxa were observed in the LFe and HFe groups. The HFe group exhibited reduced *Campylobacterota* relative to the Con and LFe groups on d6, an effect persisted on d12 relative to the LFe group (*P* < 0.05). On d12, the LFe group showed enrichment of *Campylobacteria* and *Escherichia-Shigella* compared with the HFe group (*P* < 0.05). On d18, *Rikenllaceae RC9 gut group* was significantly lower in the LFe group compared with the HFe group (2.7% vs. 12.7%, *p* < 0.05). On d24, the HFe group showed decreased abundance of *Christensenellaceae R.7 group* relative to the Con group (*p* < 0.05), while the LFe diet led to a significant reduction in *Actinobacteriota* phylum (*p* < 0.05). No differences in *Lactobacillus* abundance were observed across iron treatments throughout the study.

Similar to the LFe group, *Actinobacteriota* abundance was significantly decreased in the HCu group on d18 and d24 relative to the Con group (*P* < 0.05). At the genus level, this corresponded to a complete absence of *Bifidobacterium* at both days (*P* < 0.05). *Lactobacillus* abundance was also reduced compared to the Con group on d6 (*p* < 0.05). In contrast, *Spirochaetota* abundance was significantly increased in the HCu group on d24 (*p* < 0.05), indicating that Cu-based growth promoter led to modest but significant changes in microbial composition.

## Discussion

In the current study, excessive dietary supplementation significantly increased hepatic accumulation and fecal excretion of the respective metals, confirming the effectiveness of dietary treatments. The LFe pigs had the lowest fecal iron concentration but normal Hb and Hct and similar hepatic iron concentrations as Con pigs. These signs suggested that our goal to reduce enteric iron availability without causing systemic iron deficiency or anemia was successfully achieved by combining a low-iron diet with parenteral iron injections.

### Treatment effects on growth performance and frequency of diarrhea

Previous research has suggested that the current iron recommendation may not be sufficient to maximize the growth potential of nursery pigs [32, 33]. Supplementation of a basal nursery diet (96 mg Fe/kg) with a high level of ferrous glycine chelate (2,000 mg/kg) was shown to improve ADG and ADFI [34]. In contrast, Chen et al. (2020) did not observe any growth improvement in postweaning pigs when iron supplementation (as ferrous sulfate) increased from 50 mg/kg up to 800 mg/kg [35]. Instead, higher levels of iron supplementation (500 and 800 mg/kg) were associated with elevated fecal scores, suggesting an increased incidence of diarrhea [35]. Despite the lack of a growth-enhancing effect, HFe diet (1219 mg/kg) reduced the frequency of diarrhea. It should be noted that, in the current study, overall diarrheal illness after ETEC inoculation was moderate compared with our previous studies using the same ETEC strain. Inconsistent effects on diarrhea have also been reported at lower levels of iron supplementation. Lee et al. (2008) observed a linear increase in diarrhea incidence with incremental increases in supplemental iron from 0 to 250 mg/kg [36], whereas Gao et al. (2025) reported a 40% reduction in diarrhea incidence when a basal nursery diet (100 mg Fe/kg) was supplemented with 50 mg Fe/kg as ferrous glycine [37]. Taken together, these varied responses cannot be fully attributed to differences in initial iron or hematological status. The involvement of contextual factors (e.g., ingredient composition of basal diet) is plausible and warrants future investigation.

Pharmacological levels of Cu (125–250 mg/kg) supplementation in nursery diets have shown growth-promoting and diarrhea-reducing effects in postweaning pigs [38–40]. While the underlying mechanisms are yet unclear, these beneficial effects have been generally attributed to the antimicrobial properties of Cu, altered mineral interactions, and increased nutrient digestibility through enhancing pancreatic enzyme activities despite some inconsistency across studies [41–43]. Similar to the HFe group, HCu pigs exhibited lower frequency of diarrhea. The HCu treatment, however, did not enhance postweaning growth in the current study. Furthermore, the lowest Hb, Hct, and tissue iron concentrations observed in HCu pigs suggested a negative impact of excess Cu intake on iron utilization and hematological status. Others also reported similar findings [44, 45], underscoring the need of additional iron supplementation to offset the negative effects of excess Cu.

The pharmacological levels of ZnO have been well-studied for its effectiveness in promoting growth and preventing infection and diarrhea in postweaning pigs [46, 47], it is unexpected that HZn treatment did not improve growth parameters in this study. One potential explanation for the lack of differences in growth observed in pigs received metal growth promoters may relate to the superior nutritional quality of the experimental diets. Although it is not uncommon to include milk products in early post-weaning diets (e.g., phase 1 nursery diets), the level (~ 35%) of dried whey and non-fat dry milk in our experimental diets were much higher than that used in commercial nursery diets. It is plausible that the high-quality experimental diets help alleviate weaning-induced enteric distress and mask the growth-promoting effects of HZn and HCu treatments. Notably, the growth promoting effects of ZnO and copper are most often reported in weaned piglets fed corn and soybean meal diets or barley-wheat-soybean meal diets containing less milk products [38, 48–50]. Previously, Araujo et al. (2010) found that piglets weaned at 21 days of age and fed a corn- and soybean-based diet had lower weight gain than piglets fed diets where nonfat dry milk powder was included [51]. Future research should evaluate the interaction effects of metal-based growth promoters and the quality of basal diet on growth performance and diarrhea frequency.

### Treatment effects on the gut microbiome

Like most organisms, bacteria often require iron for various metabolic processes that are fundamental to survival. In particular, many pathogenic bacteria, such as *Staphylococcus* and certain *E. coli* strains, are facultative anaerobes with robust capabilities to sequester iron during colonization [52, 53]. Highly conserved, iron-responsive transcription factors, for example, allow bacteria to respond to iron deprivation and regulate the expression of iron acquisition mechanisms, such as siderophore or hemophore production [53]. To prevent infection, the host tightly regulates iron availability through a process known as nutritional immunity, thus creating competition between bacteria and the host [54]. Iron supplementation or fortification has been shown to modulate the gut microbiome as well as alter the risk for diarrheal disease [55, 56]. These outcomes, however, are inconsistent as they may be context dependent [57]. As such, thorough investigation of the gut microbiota is required when modulating luminal iron availability.

Reducing luminal iron was associated with a gradual constraint on microbial expansion. While differences between treatment groups were negligible at individual timepoints, the divergence observed over time suggests that dietary iron restriction may gradually influence the community trajectory rather than inducing an abrupt compositional shift. These findings agree with that of Coe et al. (2021), where reducing luminal iron in C57BL/6 mice for two weeks led to a decrease in alpha diversity [58]. Although the role of the microbiome in ETEC susceptibility is not fully understood, colonization resistance has been linked to increased microbial diversity and abundance [59, 60]. Conversely, ETEC infection was associated with significant reduction in jejunal and fecal microbial diversity in piglets [61, 62].

Taxonomic analysis revealed only modest community restructuring in the LFe and HFe diets. However, early enrichment of *Escherichia-Shigella* and *Camplylobacteria* under low iron conditions suggests that reducing luminal iron may transiently favor taxa associated with enteric disease risk. The combination of low microbial complexity and the presence of species closely related to the pathogen has been linked to reduced colonization resistance [63]. Given the prevalence of ETEC during the first two weeks post-weaning, the ability of *Escherichia-Shigella* to thrive in low iron environments during the early development of the microbiome may increase susceptibility to infection [64]. Although the alteration in abundance was not sustained past d12, these results are in agreement with previous data observed in animal models of iron deficiency. Iron deficient diets have been shown to significantly increase the abundance of lactobacilli and *Enterobacteriaeae* in iron deprived mice and young Sprague Dawley rats [65, 66]. Members of lactobacilli are known iron-independent bacteria that exert beneficial effects on intestinal health through many mechanisms including the promotion of mucus secretion, secreting anti-microbial substances, and the prevention of pathogen adherence [67]. Furthermore, in a study by Knight et al. (2019), the colonic and fecal microbiome of iron deficient piglets were enriched with *Lactobacillus* and *Bifidobacterium* [68]. Given that *Escherichia-Shigella* and Campylobacteria abundance in the LFe group were elevated in the absence of *Lactobacillus* enrichment, the current study raises questions regarding the inferred benefits of a low iron diet. Together, the restructuring of the microbiome observed in the LFe group may indicate increased risk for ETEC colonization, highlighting the need for further investigation into the role of iron in shaping the microbiome.

In contrast, the HFe group consistently maintained minimal to no changes in the microbiome throughout the duration of the study. Previously, iron supplementation in human and rodent studies have shown to reduce beneficial bacteria, such as *Bifidobacterium* and *Lactobacillus* and increase *Enterobacteriaceae* [69–71]. In a recent study involving postweaning pigs, however, increases in ferrous sulfate supplementation increased the abundance of *L. amylovorus* without altering *E. coli* and bifidobacteria populations [35]. Despite these findings, the current study did not detect clear signs of iron-induced changes to microbial composition. In conjunction with the reduction of total days of diarrhea, this suggests that the mechanism in which HFe may reduce diarrhea may be associated with alterations in host or bacterial metabolism rather than shifts in the microbiome.

The impact of pharmacological doses of ZnO on the intestinal microbiota remains controversial as several studies have report minor alterations, whereas others have reported dynamic community restructuring. Growth-promoting effect of ZnO has been largely attributed to its ability to induce oxidative stress in bacteria, thus altering bacterial growth and metabolism [72]. This effect, however, may also be influenced by host-mediated mechanisms such as alterations in gastrointestinal pH, intestinal barrier function, and mucosal immune response [72, 73]. While some have reported decreased microbial diversity, such as decreases in *Enterobacteriaceae*, others have found no effect on bacterial populations [74–76]. It has also been suggested, however, that pharmaceutical doses of ZnO may increase the number of enterobacteria and general bacterial diversity [77, 78]. Additional reports have shown that ZnO may enrich beneficial bacteria such as *Lactobacillaceae* and increase the *Lactobacillus*-to-*E. coli* ratio within the ileum [79, 80].

In the current study, the HZn diet led to the largest changes in the gut microbiota, driven by early reductions microbial richness. The observed changes in community structure remained relatively stable throughout the study, consistent with reports suggesting that ZnO may limit dysbiosis by reducing microbial diversity and stabilizing composition and functionality [81–83]. Evaluation of distance metrics indicated that the early restructuring was driven by shifts in phylogenetically distinct taxa rather than changes in relative abundance alone. The taxonomic restructuring observed in the HZn group was characterized by the enrichment of *Bacteroidota* alongside the reduction of others such as *Ruminoccocus*, *Treponema*, and *Rikenellaceae RC9 gut group*. The *Bacteroidota* phylum has been recognized for it’s ability to produce a wide range of carbohydrate-active enzymes (CAZymes), allowing for the degradation of complex carbohydrates such as polysaccharides thus potentially enhancing SCFA production [84]. Given that piglets with ETEC infection have been shown to have a lower *Bacteroidota*:*Bacillota* ratio in the jejunum and feces, the observed enrichment of *Bacteroidota* suggests enhanced gastrointestinal function and reduced risk for diarrheal disease [61].

Similar to HFe group, the HCu group also induced minimal changes in overall the gut microbial diversity. Of the few changes observed, the HCu diet induced reductions in *Bifidobacterium* abundance. Although this finding aligns with prior work in pigs and poultry, others have reported broader copper-induced restructuring of the microbiome [85–87]. Previously, supplementation with 300 mg/kg of copper was shown to alter the abundance of bacteria in the *Clostridia* genera and reduced the abundance of butyrate producing bacteria such as *Coprococcus*, *Roseburia*, and *Acidaminococcus* in suckling piglets [88]. It has also been reported that 250 mg/kg of CuSO_4_ in weaned pigs can reduce cecum microbial diversity and decrease *Enterobacteriaceae*, *lactobacilli*, and *Streptococcus* abundances [41, 85, 89]. These effects have been attributed to copper’s ability to participate in a Fenton-like reaction, thus inducing oxidative stress through lipoperoxidation and DNA damage. The findings of the current study align with previous work on copper supplementation revealing no differences in overall diversity, but affected the composition of microbial communities between treatment groups [89]. The observed reduction in *Bifidobacterium* abundance suggests that copper may influence specific taxa without major disruptions to microbial diversity.

## Conclusions

This study showed that dietary iron and alternative growth promoters exerted varied influences on gut microbial ecology and diarrheal outcomes. While none of the treatments improved growth performance compared to the Con group, high levels of dietary iron and copper reduced total days of diarrhea during the study. Pharmacological ZnO induced the most pronounced and persistent changes in gut microbiome, characterized by reduced diversity and *Bacteroidota* dominance, suggesting distinct selective pressure on microbial communities. Conversely, low dietary iron promoted the early expansion of *Escherichia-Shigella*, highlighting a potential risk of increased susceptibility to enteric pathogens. Collectively, these findings suggest that iron and copper supplementation may mitigate post-weaning diarrhea without major microbiome disruption, whereas ZnO may stabilize microbial composition but at the expense of reduced diversity. Future studies should utilize industry-relevant corn–soybean meal diets and evaluate the relationship between fecal pathogen load to gut microbiota diversity to determine the role of these dietary trace minerals in pathogen susceptibility and gut health.

## Supplementary Information

Below is the link to the electronic supplementary material.

Supplementary Material 1

## Data Availability

Reads were deposited into the NCBI Sequencing Read Archive (SRA) (BioProject: PRJNA1330844). The 16 S rRNA sequencing data have been deposited in the NCBI Sequence Read Archive database under BioProject ID PRJNA1330844. The project information is accessible at the following link: (https://www.ncbi.nlm.nih.gov/sra/PRJNA1330844).

## References

[1] Zimmerman JJ. Diseases of swine. Eleventh edn. Hoboken, NJ: Wiley Blackwell; 2019.

[2] Paiva RC, Burrough ER, Macedo N, Silva A, de Lagarde M, Fairbrother JM, Piñeiro PE, Almeida MN. Description of a contemporary pathogenic Escherichia coli isolated from pigs with post-weaning diarrhea in the United States from 2010 to 2023. Vet Res. 2025;56:130. 40597240 10.1186/s13567-025-01568-y PMC12218006

[3] Canibe N, Højberg O, Kongsted HA-O, Vodolazska D, Lauridsen CA-O, Nielsen TA-O. Schönherz AA-O. Review on preventive measures to reduce post-weaning diarrhoea in piglets. Animals. 2022;12:2585. 10.3390/ani12192585 10.3390/ani12192585 PMC9558551 36230326

[4] Shurson GA-O, Urriola PA-O, Hung YT. Too much of a good thing: rethinking feed formulation and feeding practices for zinc in swine diets to achieve one health and environmental sustainability. Animals. 2022;12:3374. 10.3390/ani12233374 10.3390/ani12233374 PMC9739216 36496895

[5] Ng’ang’aZW, Tous N, Ballester M, Leskovec J, Jimenez-Moya B, Beltrán-Debón R, Torrallardona D, Tarradas J. Impact of zinc oxide on gut health, immunity, and growth in weaned piglets: exploring potential modes of action. Front Vet Sci. 2025;12:1645900. 10.3389/fvets.2025.1645900 10.3389/fvets.2025.1645900 PMC12434765 40959845

[6] SeilerC, Berendonk TU. Heavy metal driven co-selection of antibiotic resistance in soil and water bodies impacted by agriculture and aquaculture. Front Microbiol. 2012;3:399. 10.3389/fmicb.2012.00399 23248620 10.3389/fmicb.2012.00399 PMC3522115

[7] CiesinskiLA-O, Guenther S, Pieper R, Kalisch M, Bednorz C, Wieler LH. High dietary zinc feeding promotes persistence of multi-resistant E. coli in the swine gut. PLoS One. 2018;13:e0191660. 10.1371/journal.pone.0191660 10.1371/journal.pone.0191660 PMC5786291 29373597

[8] Gillieatt BF, Coleman NA-O. Unravelling the mechanisms of antibiotic and heavy metal resistance co-selection in environmental bacteria. FEMS Microbiol Rev. 2024;48:fuae017. 10.1093/femsre/fuae017 10.1093/femsre/fuae017 PMC11253441 38897736

[9] Chevalier TB, Adeola O, Carter SD, Dove CR, Estienne MJ, Levesque CL, Maxwell CV, Tsai T, Lindemann MD. A cooperative study assessing the effects of a second iron injection administered before weaning on growth performance, hematological status, and tissue mineral concentrations of nursery pigs*. Appl Anim Sci. 2024;40:112–23.

[10] National Research Council. Committee on Nutrient Requirements of S. Nutrient requirements of swine. [11th rev. ]. edn. Washington, D.C: National Academies; 2012.

[11] Frawley ER, Fang FC. The ins and outs of bacterial iron metabolism. Mol Microbiol. 2014;93:609–16. 25040830 10.1111/mmi.12709 PMC4135372

[12] Page MGP. The Role of Iron and Siderophores in Infection, and the Development of Siderophore Antibiotics. Clin Infect Dis. 2019;69:S529–37. 31724044 10.1093/cid/ciz825 PMC6853763

[13] Coe GL, Pinkham NV, Celis AI, Johnson C, DuBois JL, Walk SA-O. Dynamic gut microbiome changes in response to low-iron challenge. Appl Environ Microbiol. 2021;87:e02307-20. 10.1128/AEM.02307-20 10.1128/AEM.02307-20 PMC7848906 33188000

[14] McMillen SA-O, Thomas S, Liang E, Nonnecke EB, Slupsky CA-O, Lönnerdal B. Gut microbiome alterations following postnatal iron supplementation depend on iron form and persist into adulthood. Nutrients. 2022;14:412 10.3390/nu14030412 10.3390/nu14030412 PMC8838803 35276770

[15] ZimmermannMB, Chassard C, Fau - Rohner F, Rohner F, Fau - C, Nindjin C, Fau - Dostal A, Dostal A, Fau - Utzinger J, Utzinger J, Fau - Ghattas H, Ghattas H Fau - Lacroix C, Lacroix C Fau - Hurrell RF, Hurrell RF. The effects of iron fortification on the gut microbiota in African children: a randomized controlled trial in Cote d’Ivoire. Am J Clin Nutr. 2010;92:1406–15. 10.3945/ajcn.110.004564 10.3945/ajcn.110.004564 20962160

[16] EllermannM, Gharaibeh RZ, Maharshak N, Peréz-Chanona E, Jobin C, Carroll IM, Arthur JA-O, Plevy SE, Fodor AA, Brouwer CA-O, Sartor RB. Dietary iron variably modulates assembly of the intestinal microbiota in colitis-resistant and colitis-susceptible mice. Gut Microbes. 2020;11:32–50. 10.1080/19490976.2019.1599794 10.1080/19490976.2019.1599794 PMC6973310 31179826

[17] IppolitoJR, Piccolo BD, Robeson MS, Barney DE Jr., Ali J, Singh P, Hennigar SR. Iron deficient diets modify the gut microbiome and reduce the severity of enteric infection in a mouse model of S. Typhimurium-induced enterocolitis. J Nutr Biochem. 2022;107:109065. 10.1016/j.jnutbio.2022.109065 10.1016/j.jnutbio.2022.109065 35609848

[18] HainesSA-O, Arnaud-Barbe N, Poncet D, Reverchon S, Wawrzyniak J, Nasser W. Renauld-Mongénie G: IscR Regulates Synthesis of Colonization Factor Antigen I Fimbriae in Response to Iron Starvation in Enterotoxigenic Escherichia coli. J Bacteriol. 2015;197:2896–907. 10.1128/JB.00214-15 10.1128/JB.00214-15 PMC4542172 26124243

[19] Karjalainen TK, Evans Dg Fau - Evans DJ, Jr., Evans Dj Jr Fau - Graham DY, Graham Dy Fau - Lee CH, Lee CH. Iron represses the expression of CFA/I fimbriae of enterotoxigenic E. coli. Microb Pathog. 1991;11:317–23. 10.1016/0882-4010(91)90017-5 1687752

[20] Sanchez KK, Chen GY, Schieber AMP, Redford SE, Shokhirev MN, Leblanc M, Lee YM, Ayres JS. Cooperative Metabolic Adaptations in the Host Can Favor Asymptomatic Infection and Select for Attenuated Virulence in an Enteric Pathogen. Cell. 2018;175:146–e158115. 30100182 10.1016/j.cell.2018.07.016 PMC6447043

[21] Kreuzer S, Reissmann M, Brockmann GA. New fast and cost-effective gene test to get the ETEC F18 receptor status in pigs. Vet Microbiol. 2013;163:392–4. 23395292 10.1016/j.vetmic.2012.12.040

[22] Bolyen E, Rideout JR, Dillon MR, Bokulich NA, Abnet CC, Al-Ghalith GA, Alexander H, Alm EJ, Arumugam M, Asnicar F, et al. Reproducible, interactive, scalable and extensible microbiome data science using QIIME 2. In Nat Biotechnol. United States; 2019;37:852–857. 10.1038/s41587-019-0209-9 PMC7015180 31341288

[23] Callahan BJ, McMurdie PJ, Rosen MJ, Han AW, Johnson AJ, Holmes SP. DADA2: High-resolution sample inference from Illumina amplicon data. Nat Methods. 2016;13:581–3. 27214047 10.1038/nmeth.3869 PMC4927377

[24] Pedregosa F, Varoquaux G, Gramfort A, Michel V, Thirion B, Grisel O, Blondel M, Prettenhofer P, Weiss R, Dubourg V, Vanderplas J. Scikit-learn: machine learning in Python. J Mach Learn Res. 2011;12:2825–30. 10.48550/arXiv.1201.0490

[25] Quast C, Pruesse E, Yilmaz P, Gerken J, Schweer T, Yarza P, Peplies J, Glöckner FO. The SILVA ribosomal RNA gene database project: improved data processing and web-based tools. Nucleic Acids Res. 2013;41:D590–596. 23193283 10.1093/nar/gks1219 PMC3531112

[26] Bokulich NA, Kaehler BD, Rideout JR, Dillon M, Bolyen E, Knight R, Huttley GA, Gregory Caporaso J. Optimizing taxonomic classification of marker-gene amplicon sequences with QIIME 2’s q2-feature-classifier plugin. Microbiome. 2018;6:90. 29773078 10.1186/s40168-018-0470-z PMC5956843

[27] Katoh K, Standley DM. MAFFT multiple sequence alignment software version 7: improvements in performance and usability. Mol Biol Evol. 2013;30:772–80. 23329690 10.1093/molbev/mst010 PMC3603318

[28] Price MN, Dehal PS, Arkin AP. FastTree 2–approximately maximum-likelihood trees for large alignments. PLoS ONE. 2010;5:e9490. 20224823 10.1371/journal.pone.0009490 PMC2835736

[29] Shannon CE. A mathematical theory of communication. Bell Syst Tech J. 1948;27:379–423.

[30] Faith DP. Conservation evaluation and phylogenetic diversity. Biol Conserv. 1992;61:1–10.

[31] Benjamini YaHY. Controlling the False Discovery Rate: A Practical and Powerful Approach to Multiple Testing. J Roy Stat Soc: Ser B (Methodol). 1995;57:289–300.

[32] Johnson AJ, Li W, Dittrich BI, Cole AC, Prodell MK, Lyons JW, Fritz SA, Fregulia P, Chen C, Kwon CH, Jang YD. Effect of second iron injection on growth performance, hematological parameters, and fecal microbiome of piglets fed different dietary iron levels. J Anim Sci. 2025;103. 10.1093/jas/skae371 PMC11700587 39657578

[33] Deng Q, Wang Y, Wang X, Wang Q, Yi Z, Xia J, Hu Y, Zhang Y, Wang J, Wang L et al. Effects of dietary iron level on growth performance, hematological status, and intestinal function in growing-finishing pigs. J Anim Sci. 2021;99. 10.1093/jas/skab002 PMC7846194 33515478

[34] Ma J, Liu S, Piao X, Wang C, Wang J, Lin YS, Hsu TP, Liu L. Dietary Supplementation of Ferrous Glycine Chelate Improves Growth Performance of Piglets by Enhancing Serum Immune Antioxidant Properties, Modulating Microbial Structure and Its Metabolic Function in the Early Stage. Front Vet Sci. 2022;9:876965. 35548055 10.3389/fvets.2022.876965 PMC9083199

[35] Chen S, Wu X, Wang X, Shao Y, Tu Q, Yang H, Yin J, Yin Y. Responses of Intestinal Microbiota and Immunity to Increasing Dietary Levels of Iron Using a Piglet Model. Front Cell Dev Biol. 2020;8:603392. 33392192 10.3389/fcell.2020.603392 PMC7773786

[36] Lee SH, Shinde P, Choi J, Park M, Ohh S, Kwon IK, Pak SI, Chae BJ. Effects of dietary iron levels on growth performance, hematological status, liver mineral concentration, fecal microflora, and diarrhea incidence in weanling pigs. Biol Trace Elem Res. 2008;126(Suppl 1):S57–68. 18759068 10.1007/s12011-008-8209-5

[37] Gao Q, Zhang Y, Wu Y, Gu D, Chen J, Yin C, Wu H, Zhu D, Chen D, Wu A. Dietary Fe-Gly supplementation attenuates enterotoxigenic Escherichia coli (ETEC)-induced inflammation response and intestinal barrier dysfunction in piglets. Front Vet Sci. 2025;12:1537604. 39944187 10.3389/fvets.2025.1537604 PMC11814430

[38] Stahly TS, Cromwell GL, Monegue HJ. Effects of the dietary inclusion of copper and(or) antibiotics on the performance of weanling pigs. J Anim Sci. 1980;51:1347–51. 6782067 10.2527/jas1981.5161347x

[39] Bikker P, Jongbloed AW, van Baal J. Dose-dependent effects of copper supplementation of nursery diets on growth performance and fecal consistency in weaned pigs1. J Anim Sci. 2016;94:181–6.

[40] Ma YL, Zanton GI, Zhao J, Wedekind K, Escobar J, Vazquez-Añón M. Multitrial analysis of the effects of copper level and source on performance in nursery pigs. J Anim Sci. 2015;93:606–14. 25548207 10.2527/jas.2014-7796

[41] Mei S-F, Yu B, Ju C-F, Zhu D, Chen D-W. Effect of different levels of copper on growth performance and cecal ecosystem of newly weaned piglets. Italian J Anim Sci. 2010;9:e71.

[42] Luo XG, Dove CR. Effect of dietary copper and fat on nutrient utilization, digestive enzyme activities, and tissue mineral levels in weanling pigs. J Anim Sci. 1996;74:1888–96. 8856443 10.2527/1996.7481888x

[43] Salah I, Parkin IP, Allan E. Copper as an antimicrobial agent: recent advances. RSC Adv. 2021;11:18179–86. 35480904 10.1039/d1ra02149d PMC9033467

[44] Estienne MJ, Clark-Deener SG, Williams KA. Growth performance and hematology characteristics in pigs treated with iron at weaning as influenced by nursery diets supplemented with copper. J Swine Health Prod. 2020;28:190–204.

[45] Dove CR, Haydon KD. The effect of copper addition to diets with various iron levels on the performance and hematology of weanling swine. J Anim Sci. 1991;69:2013–9. 10.2527/1991.6952013x 10.2527/1991.6952013x 2066311

[46] Luise D, Negrini C, Correa F, Trevisi P. Effect and mode of action of different doses and sources of zinc in weaning pigs using a meta-analytical and systematic review approach. Italian J Anim Sci. 2024;23:241–58.

[47] Sales J. Effects of pharmacological concentrations of dietary zinc oxide on growth of post-weaning pigs: a meta-analysis. Biol Trace Elem Res. 2013;152:343–9. 10.1007/s12011-013-9638-3 10.1007/s12011-013-9638-3 23463368

[48] Espinosa CD, Fry RS, Usry JL, Stein HH. Copper hydroxychloride improves growth performance and reduces diarrhea frequency of weanling pigs fed a corn-soybean meal diet but does not change apparent total tract digestibility of energy and acid hydrolyzed ether extract. J Anim Sci. 2017;95:5447–54. 29293761 10.2527/jas2017.1702 PMC6357799

[49] Forouzandeh A, Blavi L, Pérez JF, D’Angelo M, González-Solé F, Monteiro A, Stein HH, Solà-Oriol D. How copper can impact pig growth: comparing the effect of copper sulfate and monovalent copper oxide on oxidative status, inflammation, gene abundance, and microbial modulation as potential mechanisms of action. J Anim Sci. 2022;100. 10.1093/jas/skac224 PMC9486896 35723874

[50] Sales J. Effects of Pharmacological Concentrations of Dietary Zinc Oxide on Growth of Post-weaning Pigs: A Meta-analysis. Biol Trace Elem Res. 2013;152:343–9. 23463368 10.1007/s12011-013-9638-3

[51] Silva, WAGAaASFaDRaPCBaBAN. Effects of diet protein source on the behavior of piglets after weaning. Livest Sci. 2010;132:35–40.

[52] Hammer ND, Skaar EP. Molecular mechanisms of Staphylococcus aureus iron acquisition. Annu Rev Microbiol. 2011;65:129–47. 21639791 10.1146/annurev-micro-090110-102851 PMC3807827

[53] SheldonJR, Laakso HA, Heinrichs DE. Iron acquisition strategies of bacterial pathogens. In Virulence Mechanisms of Bacterial Pathogens. 2016:43–85. 10.1128/microbiolspec.vmbf-0010-2015 10.1128/microbiolspec.VMBF-0010-2015 27227297

[54] Murdoch CC, Skaar EP. Nutritional immunity: the battle for nutrient metals at the host–pathogen interface. Nat Rev Microbiol. 2022;20:657–70. 35641670 10.1038/s41579-022-00745-6 PMC9153222

[55] Ding H, Yu X, Chen L, Han J, Zhao Y, Feng J. Tolerable upper intake level of iron damages the intestine and alters the intestinal flora in weaned piglets. Metallomics. 2020;12:1356–69. 32583831 10.1039/d0mt00096e

[56] Paganini D, Zimmermann MB. The effects of iron fortification and supplementation on the gut microbiome and diarrhea in infants and children: a review. Am J Clin Nutr. 2017;106:s1688–93. 10.3945/ajcn.117.156067 PMC5701709 29070552

[57] Dewey KG, Domellöf M, Cohen RJ, Landa Rivera L, Hernell O, Lönnerdal B. Iron supplementation affects growth and morbidity of breast-fed infants: results of a randomized trial in Sweden and Honduras. J Nutr. 2002;132:3249–55. 12421836 10.1093/jn/132.11.3249

[58] Coe Genevieve L, Pinkham Nicholas V, Celis Arianna I, Johnson C, DuBois Jennifer L. Walk Seth T: Dynamic Gut Microbiome Changes in Response to Low-Iron Challenge. Appl Environ Microbiol. 2021;87:e02307–02320. 33188000 10.1128/AEM.02307-20 PMC7848906

[59] Lawley TD, Walker AW. Intestinal colonization resistance. Immunology. 2013;138:1–11. 23240815 10.1111/j.1365-2567.2012.03616.x PMC3533696

[60] Bäumler AJ, Sperandio V. Interactions between the microbiota and pathogenic bacteria in the gut. Nature. 2016;535:85–93. 27383983 10.1038/nature18849 PMC5114849

[61] Bin P, Tang Z, Liu S, Chen S, Xia Y, Liu J, Wu H, Zhu G. Intestinal microbiota mediates Enterotoxigenic Escherichia coli-induced diarrhea in piglets. BMC Vet Res. 2018;14:385. 30518356 10.1186/s12917-018-1704-9 PMC6282381

[62] Higginson EE, Sayeed MA, Pereira Dias J, Shetty V, Ballal M, Srivastava SK, Willis I, Qadri F, Dougan G, Mutreja A. Microbiome Profiling of Enterotoxigenic Escherichia coli (ETEC) Carriers Highlights Signature Differences between Symptomatic and Asymptomatic Individuals. mBio. 2022;13:e0015722. 35536001 10.1128/mbio.00157-22 PMC9239084

[63] Stecher B, Chaffron S, Käppeli R, Hapfelmeier S, Freedrich S, Weber TC, Kirundi J, Suar M, McCoy KD, von Mering C, et al. Like will to like: abundances of closely related species can predict susceptibility to intestinal colonization by pathogenic and commensal bacteria. PLoS Pathog. 2010;6:e1000711. 20062525 10.1371/journal.ppat.1000711 PMC2796170

[64] Sun Y, Kim SW. Intestinal challenge with enterotoxigenic Escherichia coli in pigs, and nutritional intervention to prevent postweaning diarrhea. Anim Nutr. 2017;3:322–30. 29767133 10.1016/j.aninu.2017.10.001 PMC5941267

[65] Tompkins GR, O’Dell NL, Bryson IT, Pennington CB. The effects of dietary ferric iron and iron deprivation on the bacterial composition of the mouse intestine. Curr Microbiol. 2001;43:38–42. 11375662 10.1007/s002840010257

[66] Dostal A, Chassard C, Hilty FM, Zimmermann MB, Jaeggi T, Rossi S, Lacroix C. Iron depletion and repletion with ferrous sulfate or electrolytic iron modifies the composition and metabolic activity of the gut microbiota in rats. J Nutr. 2012;142:271–7. 22190022 10.3945/jn.111.148643 PMC3260059

[67] Dempsey E, Corr SC. Lactobacillus spp. for Gastrointestinal Health: Current and Future Perspectives. Front Immunol. 2022;13:840245. 35464397 10.3389/fimmu.2022.840245 PMC9019120

[68] Knight LC, Wang M, Donovan SM, Dilger RN. Early-Life Iron Deficiency and Subsequent Repletion Alters Development of the Colonic Microbiota in the Pig. Front Nutr. 2019;6:120. 31440513 10.3389/fnut.2019.00120 PMC6692694

[69] Mevissen-Verhage EA, Marcelis JH, Harmsen-Van Amerongen WC, de Vos NM, Verhoef J. Effect of iron on neonatal gut flora during the first three months of life. Eur J Clin Microbiol. 1985;4:273–8. 3894015 10.1007/BF02013651

[70] Zimmermann MB, Chassard C, Rohner F, N’Goran EK, Nindjin C, Dostal A, Utzinger J, Ghattas H, Lacroix C, Hurrell RF. The effects of iron fortification on the gut microbiota in African children: a randomized controlled trial in Cote d’Ivoire. Am J Clin Nutr. 2010;92:1406–15. 20962160 10.3945/ajcn.110.004564

[71] Jaeggi T, Kortman GA, Moretti D, Chassard C, Holding P, Dostal A, Boekhorst J, Timmerman HM, Swinkels DW, Tjalsma H, et al. Iron fortification adversely affects the gut microbiome, increases pathogen abundance and induces intestinal inflammation in Kenyan infants. Gut. 2015;64:731–42. 25143342 10.1136/gutjnl-2014-307720

[72] Tang X, Xiong K, Zeng Y, Fang R. The mechanism of zinc oxide in alleviating diarrhea in piglets after weaning: a review from the perspective of intestinal barrier function. Int J Mol Sci. 2024;25. 10.3390/ijms251810040 PMC11432186 39337525

[73] Hu CH, Song ZH, Xiao K, Song J, Jiao le F, Ke YL. Zinc oxide influences intestinal integrity, the expressions of genes associated with inflammation and TLR4-myeloid differentiation factor 88 signaling pathways in weanling pigs. Innate Immun. 2014;20:478–86. 23956359 10.1177/1753425913499947

[74] Yu T, Zhu C, Chen S, Gao L, Lv H, Feng R, Zhu Q, Xu J, Chen Z, Jiang Z. Dietary High Zinc Oxide Modulates the Microbiome of Ileum and Colon in Weaned Piglets. Front Microbiol. 2017;8:825. 28536569 10.3389/fmicb.2017.00825 PMC5422713

[75] Starke IC, Pieper R, Neumann K, Zentek J, Vahjen W. The impact of high dietary zinc oxide on the development of the intestinal microbiota in weaned piglets. FEMS Microbiol Ecol. 2014;87:416–27. 24118028 10.1111/1574-6941.12233

[76] Li BT, Kessel AGV, Caine WR, Huang SX, Kirkwood RN. Small intestinal morphology and bacterial populations in ileal digesta and feces of newly weaned pigs receiving a high dietary level of zinc oxide. Can J Anim Sci. 2001;81:511–6.

[77] Pieper, RaVWaNKaVKAGaZJ. Dose-dependent effects of dietary zinc oxide on bacterial communities and metabolic profiles in the ileum of weaned pigs. J Anim Physiol Anim Nutr. 2012;96:825–33. 10.1111/j.1439-0396.2011.01231.x 21929727

[78] Vahjen W, Pieper R, Zentek J. Increased dietary zinc oxide changes the bacterial core and enterobacterial composition in the ileum of piglets. J Anim Sci. 2011;89:2430–9. 21383037 10.2527/jas.2010-3270

[79] Shen J, Chen Y, Wang Z, Zhou A, He M, Mao L, Zou H, Peng Q, Xue B, Wang L, et al. Coated zinc oxide improves intestinal immunity function and regulates microbiota composition in weaned piglets. Br J Nutr. 2014;111:2123–34. 24606984 10.1017/S0007114514000300

[80] Christensen B, Zhu C, Mohammadigheisar M, Schulze H, Huber LA, Kiarie EG. Growth performance, immune status, gastrointestinal tract ecology, and function in nursery pigs fed enzymatically treated yeast without or with pharmacological levels of zinc. J Anim Sci. 2022;100. 10.1093/jas/skac094 PMC9047176 35323958

[81] Ortiz Sanjuán JM, Argüello H, Cabrera-Rubio R, Crispie F, Cotter PD, Garrido JJ, Ekhlas D, Burgess CM, Manzanilla EG. Effects of removing in-feed antibiotics and zinc oxide on the taxonomy and functionality of the microbiota in post weaning pigs. Anim Microbiome. 2024;6:18. 38627869 10.1186/s42523-024-00306-7 PMC11022352

[82] Dai F, Zhao F, Huang X, Jin M, Zhou Q, Lin T, Zuo J, Zhu Y. Effects of replacing zinc oxide with different levels of zinc lactate on growth performance, serum indexes, intestinal health and gut microbiota in weaned piglets. Front Microbiol. 2025;16. 10.3389/fmicb.2025.1622700 PMC12289610 40718813

[83] Ortiz Sanjuán Juan M, Manzanilla Edgar G, Cabrera-Rubio R, Crispie F, Cotter Paul D, Garrido Juan J, Argüello H. Using Shotgun Sequencing to Describe the Changes Induced by In-Feed Zinc Oxide and Apramycin in the Microbiomes of Pigs One Week Postweaning. Microbiol Spectr. 2022;10:e01597–01522. 35950862 10.1128/spectrum.01597-22 PMC9431492

[84] McKee LSaLRSLaWBaEVGaPPBaLJ. Polysaccharide degradation by the Bacteroidetes: mechanisms and nomenclature. Environ Microbiol Rep. 2021;13:559–81. 34036727 10.1111/1758-2229.12980

[85] Brinck JE, Lassen SB, Forouzandeh A, Pan T, Wang YZ, Monteiro A, Blavi L, Solà-Oriol D, Stein HH, Su JQ, Brandt KK. Impacts of dietary copper on the swine gut microbiome and antibiotic resistome. Sci Total Environ. 2023;857:159609. 36273560 10.1016/j.scitotenv.2022.159609

[86] Muurinen J, Richert J, Wickware CL, Richert B, Johnson TA. Swine growth promotion with antibiotics or alternatives can increase antibiotic resistance gene mobility potential. Sci Rep. 2021;11:5485. 33750827 10.1038/s41598-021-84759-9 PMC7970892

[87] Sizentsov AN, Kvan OV, Miroshnikova EP, Gavrish IA, Serdaeva VA, Bykov AV. Assessment of biotoxicity of Cu nanoparticles with respect to probiotic strains of microorganisms and representatives of the normal flora of the intestine of broiler chickens. Environ Sci Pollut Res. 2018;25:15765–73. 10.1007/s11356-018-1761-4 29582323

[88] Zhang F, Zheng W, Xue Y, Yao W. Suhuai suckling piglet hindgut microbiome-metabolome responses to different dietary copper levels. Appl Microbiol Biotechnol. 2019;103:853–68. 30535578 10.1007/s00253-018-9533-0 PMC6373200

[89] Zhang Y, Zhou J, Dong Z, Li G, Wang J, Li Y, Wan D, Yang H, Yin Y. Effect of Dietary Copper on Intestinal Microbiota and Antimicrobial Resistance Profiles of Escherichia coli in Weaned Piglets. Front Microbiol. 2019;10:2808. 31921011 10.3389/fmicb.2019.02808 PMC6927916

