# Supplementary material for: Dietary iron and metal-based growth differentially modulate growth and gut microbiome of weaned piglets
Source: Anim Microbiome. 2026 Apr 11;8:64. doi: 10.1186/s42523-026-00561-w (PMC13185270; doi:10.1186/s42523-026-00561-w)
Supplement: Supplementary file 1 — Supplementary Material 1 [file 42523_2026_561_MOESM1_ESM.docx]

**Table S1.** Ingredient composition and mineral concentrations of experimental diets.

| **Ingredient** | **LFe** | **Con** | **HFe** | **HCu** | **HZn** |
| --- | --- | --- | --- | --- | --- |
| *Diet ingredients (as fed basis), %* |  |  |  |  |  |
| Corn meal | 57.13 | 57.13 | 56.73 | 57.13 | 56.82 |
| Whey, dried | 5.40 | 5.40 | 5.40 | 5.40 | 5.40 |
| Non-fat dry milk | 30.00 | 30.00 | 30.00 | 30.00 | 30.00 |
| Calcium carbonate | 0.55 | 0.55 | 0.55 | 0.55 | 0.55 |
| Dicalcium phosphate | 0.65 | 0.65 | 0.65 | 0.65 | 0.65 |
| Lysine HCL | 0.31 | 0.31 | 0.31 | 0.31 | 0.31 |
| DL-Met | 0.14 | 0.14 | 0.14 | 0.14 | 0.14 |
| Threonine | 0.10 | 0.10 | 0.10 | 0.10 | 0.10 |
| L-Tryptophan | 0.02 | 0.02 | 0.02 | 0.02 | 0.02 |
| Soybean oil | 1.00 | 1.00 | 1.00 | 1.00 | 1.00 |
| Salt | 0.40 | 0.40 | 0.40 | 0.40 | 0.40 |
| Soy protein concentrate | 4.00 | 4.00 | 4.00 | 4.00 | 4.00 |
| Mineral-vitamin premix | 0.30 | 0.30 | 0.70 | 0.30 | 0.30 |
| ZnO | 0 | 0 | 0 | 0 | 0.31 |
| Total | 100.00 | 100.00 | 100.00 | 100.00 | 100.00 |
| *Mineral-vitamin premix ingredients, g/100 kg diet* |  |  |  |  |  |
| 10×Vitamin premix^1^ | 30.00 | 30.00 | 30.00 | 30.00 | 30.00 |
| Copper sulfate pentahydrate | 7.07 | 7.07 | 7.07 | 98.23 | 7.07 |
| Ferrous sulfate heptahydrate | 0.00 | 59.74 | 597.39 | 59.74 | 59.74 |
| Manganese sulfate monohydrate | 9.23 | 9.23 | 9.23 | 9.23 | 9.23 |
| Zinc sulfate heptahydrate | 54.98 | 54.98 | 54.98 | 54.98 | 54.98 |
| Sodium selenite | 0.07 | 0.07 | 0.07 | 0.07 | 0.07 |
| Calcium iodate | 0.09 | 0.09 | 0.09 | 0.09 | 0.09 |
| corn meal | 198.56 | 138.83 | 0.00 | 47.66 | 138.83 |
| Total | 300.00 | 300.00 | 698.83 | 300.00 | 300.00 |
| *Cu, Fe, or Zn from Premix, mg/kg diet* |  |  |  |  |  |
| Cu | 18 | 18 | 18 | 250 | 18 |
| Fe | 0 | 120 | 1200 | 120 | 120 |
| Zn | 125 | 125 | 125 | 125 | 125 |
| *Total Cu, Fe, and Zn in diet, mg/kg diet (as fed basis)^2^* |  |  |  |  |  |
| Cu | 25 | 25 | 25 | 257 | 25 |
| Fe | 19 | 139 | 1219 | 139 | 139 |
| Zn | 141 | 141 | 141 | 141 | 2631 |

^1^10×Vitamin premix contains (minimum concentration): biotin (1,433 mg/kg), folic acid (5291 mg/kg), niacin (147,159 mg/kg), pantothenic acid (78,485 mg/kg), pyridoxine (772 mg/kg), riboflavin (21,936 mg/kg), vitamin A (3.71×10^7^ IU/kg), vitamin D (8.36×10^6^ IU/kg), vitamin E (220,573 IU/kg), vitamin K (4,630 mg/kg), iron (403 mg/kg), manganese (64 mg/kg), calcium (11 mg/kg).

^2^ Feed ingredients were analyzed for Cu, Fe, and Zn using atomic absorption spectrometry. Mineral levels in the experimental diets were calculated by summing the analyzed ingredient concentrations and the supplemental minerals from the premix.

**Table S2.** Observed mineral concentrations (µmol/g) of the liver, spleen, and heart on d24.

| **Tissue** | **Mineral** | **Con** | **LFe** | **HFe** | **HCu** | **HZn** |
| --- | --- | --- | --- | --- | --- | --- |
| Liver | Iron | 0.78±0.46^b^ | 0.99±0.37^b^ | 1.68±0.44^a^ | 0.60±0.38^b^ | 0.95±0.37^b^ |
|  | Copper | 0.23±0.13^b^ | 0.21±0.09^b^ | 0.18±0.06^b^ | 1.84±0.49^a^ | 0.15±0.07^b^ |
|  | Zinc | 1.15±0.39^b^ | 0.94±0.29^b^ | 1.14±0.38^b^ | 1.10±0.25^b^ | 14.28±2.41^a^ |
|  | Manganese | 0.045±0.011 | 0.044±0.004 | 0.042±0.006 | 0.042±0.005 | 0.038±0.008 |
| Spleen | Iron | 3.71±0.69^ab^ | 3.86±1.44^ab^ | 4.95±1.18^a^ | 2.66±1.46^b^ | 5.37±1.86^a^ |
|  | Copper | 0.013±0.002^bc^ | 0.014±0.002^bc^ | 0.015±0.001^b^ | 0.018±0.003^a^ | 0.012±0.001^c^ |
|  | Zinc | 0.25±0.02 | 0.25±0.03 | 0.25±0.03 | 0.27±0.03 | 0.24±0.04 |
|  | Manganese | 0.004±0.001^ab^ | 0.004±0.001^ab^ | 0.003±0.001^b^ | 0.004±0.001^a^ | 0.003±0.001^ab^ |
| Heart | Iron | 0.50±0.08^a^ | 0.45±0.07^ab^ | 0.50±0.04^a^ | 0.38±0.10^b^ | 0.50±0.12^a^ |
|  | Copper | 0.039±0.004 | 0.037±0.003 | 0.039±0.005 | 0.038±0.004 | 0.039±0.006 |
|  | Zinc | 0.26±0.02 | 0.27±0.03 | 0.26±0.02 | 0.27±0.02 | 0.29±0.02 |
|  | Manganese | 0.004±0.001^ab^ | 0.004±0.001^a^ | 0.003±0.001^b^ | 0.004±0.001^a^ | 0.004±0.001^ab^ |

Values are presented as mean ± SD. Means within a row that do not share a superscript letter (a-c) are significantly different (*P* < 0.05).

Con, Control diet; LFe, Low Iron Diet; HFe, High Iron diet; HCu, High Copper diet; HZn, High Zinc diet

**Table S3.** Relative abundances of phyla at d1, 6, 12, 15, 18, and 24

| Phylum | Day | Control | LFe | HFe | HCu | HZn |
| --- | --- | --- | --- | --- | --- | --- |
| *Bacteroidota* | 1 | 36.5 ± 14.2 | 46.3 ± 19.6 | 49.8 ± 17.4 | 55.9 ± 22.4 | 45.5 ± 15.6 |
|  | 6 | 37.6 ± 18.4 | 43.3 ± 15.7 | 37 ± 10.3 | 30.8 ± 16 | 56.7 ± 23.6 |
|  | 12 | 48.5 ± 15.9 | 49 ± 16 | 56.7 ± 14.9 | 53.2 ± 14.3 | 80.9 ± 12.7 |
|  | 15 | 56.6 ± 10.2 | 68.4 ± 18.3 | 62 ± 13.4 | 68.9 ± 9.1 | 83.5 ± 10.9 |
|  | 18 | 63.7 ± 19.6 | 65.3 ± 14.2 | 67.6 ± 13.6 | 66.4 ± 12.1 | 90.8 ± 7.4 |
|  | 24 | 61.8 ± 18.5 | 58.5 ± 24.3 | 69.8 ± 9.4 | 67.2 ± 13.8 | 85.5 ± 11.8 |
| *Firmicutes* | 1 | 28.8 ± 23.5 | 25.6 ± 14.9 | 27.9 ± 21 | 23.1 ± 21.7 | 23.1 ± 11.9 |
|  | 6 | 21.5 ± 14.4 | 20.6 ± 9.3 | 25.3 ± 7.3 | 19.4 ± 10.5 | 24.8 ± 10.2 |
|  | 12 | 18.5 ± 9.6 | 16.4 ± 6.9 | 16.8 ± 5.4 | 16.5 ± 4.2 | 12.6 ± 9.7 |
|  | 15 | 27.2 ± 9.1 | 14.4 ± 8.3 | 21.5 ± 14.5 | 13.5 ± 4.1 | 7.5 ± 3.8 |
|  | 18 | 21.7 ± 11.4 | 18.4 ± 5.4 | 17.2 ± 9.1 | 12.8 ± 5.6 | 5.6 ± 6.1 |
|  | 24 | 20 ± 15.8 | 25.8 ± 17.9 | 18.2 ± 9.8 | 18.2 ± 7.1 | 10.7 ± 8.3 |
| *Spirochaetota* | 1 | 10.7 ± 14.4 | 8.2 ± 7.1 | 2.7 ± 4.7 | 3.5 ± 4.9 | 5.2 ± 10.8 |
|  | 6 | 18.1 ± 21.4 | 24.1 ± 13.5 | 21.1 ± 13.9 | 28.4 ± 26.6 | 5.6 ± 10.9 |
|  | 12 | 17.3 ± 12.3 | 12.2 ± 12.8 | 16 ± 13.8 | 15.3 ± 10 | 0 ± 0.1 |
|  | 15 | 7 ± 7.8 | 5 ± 6.4 | 4.1 ± 4.1 | 6.1 ± 7.5 | 0 ± 0.1 |
|  | 18 | 3.4 ± 3.1 | 9.6 ± 10.2 | 7.9 ± 8.4 | 8.9 ± 9.1 | 0 ± 0 |
|  | 24 | 1.1 ± 1 | 5.8 ± 10.2 | 4.2 ± 3.3 | 6 ± 9.9 | 0 ± 0 |
| *Proteobacteria* | 1 | 11 ± 10.8 | 7.7 ± 8.2 | 6.9 ± 5.8 | 6.2 ± 6.9 | 10 ± 7.4 |
|  | 6 | 12.4 ± 11.8 | 3.6 ± 2.8 | 6.8 ± 9.9 | 13.8 ± 18.1 | 10 ± 12.4 |
|  | 12 | 6.2 ± 10.1 | 16.7 ± 20.9 | 6.1 ± 5.4 | 10.1 ± 12.6 | 1.6 ± 3 |
|  | 15 | 3.3 ± 4 | 6.4 ± 7.7 | 6.2 ± 10.4 | 6.7 ± 5.1 | 3.1 ± 3.5 |
|  | 18 | 2.3 ± 2.5 | 2.6 ± 2.4 | 2 ± 2.1 | 7.9 ± 9.1 | 0.7 ± 0.9 |
|  | 24 | 4.8 ± 5.6 | 6.8 ± 8.7 | 2.4 ± 3.2 | 5.7 ± 9.3 | 0.3 ± 0.4 |
| *Actinobacteriota* | 1 | 3.3 ± 6.6 | 2.6 ± 6.3 | 2.8 ± 5.9 | 1.1 ± 1.2 | 4.4 ± 6.9 |
|  | 6 | 2 ± 3.3 | 1.2 ± 2.4 | 1.2 ± 1.1 | 1 ± 0.8 | 0.8 ± 1.2 |
|  | 12 | 2 ± 1.8 | 1.8 ± 2.2 | 0.8 ± 0.5 | 0.9 ± 1 | 2.5 ± 2.9 |
|  | 15 | 1.2 ± 0.7 | 2.1 ± 2.5 | 1.6 ± 1.4 | 1.7 ± 1.6 | 5.6 ± 11.4 |
|  | 18 | 5.3 ± 8.4 | 0.9 ± 0.7 | 0.6 ± 0.6 | 0.5 ± 0.5 | 2.7 ± 1.7 |
|  | 24 | 6.6 ± 12.2 | 1.1 ± 2.2 | 1.7 ± 2.6 | 0.7 ± 0.9 | 3.5 ± 3.5 |
| *Desulfobacterota* | 1 | 2.3 ± 2 | 2.4 ± 1.2 | 3.1 ± 2.8 | 3.8 ± 3 | 3.5 ± 1.1 |
|  | 6 | 3.7 ± 5.2 | 1.3 ± 1 | 3 ± 3.2 | 1.2 ± 1.1 | 0.6 ± 1.2 |
|  | 12 | 2.3 ± 2.7 | 0.5 ± 0.8 | 1.1 ± 1.3 | 1.1 ± 1.3 | 0 ± 0 |
|  | 15 | 3.6 ± 3.6 | 0.9 ± 1.5 | 2.6 ± 2.6 | 1.4 ± 1.6 | 0 ± 0 |
|  | 18 | 2 ± 1.7 | 1.3 ± 0.9 | 1.5 ± 1.9 | 1 ± 1.2 | 0 ± 0 |
|  | 24 | 1.2 ± 1.2 | 1.2 ± 2 | 1.9 ± 1.4 | 1.1 ± 1.4 | 0 ± 0 |
| *Euryarchaeota* | 1 | 2.1 ± 2.3 | 3.5 ± 2.9 | 3 ± 3.5 | 3.5 ± 4.2 | 3.4 ± 2.4 |
|  | 6 | 2.6 ± 1.9 | 3 ± 4.2 | 2.6 ± 1.5 | 3.2 ± 4.2 | 0.7 ± 0.8 |
|  | 12 | 0.6 ± 0.7 | 0.1 ± 0.1 | 0.8 ± 0.7 | 0.4 ± 0.6 | 0.3 ± 0.8 |
|  | 15 | 0 ± 0 | 0 ± 0 | 0 ± 0 | 0 ± 0 | 0 ± 0 |
|  | 18 | 0 ± 0 | 0 ± 0 | 0 ± 0 | 0 ± 0 | 0 ± 0 |
|  | 24 | 0.7 ± 2 | 0 ± 0 | 0 ± 0 | 0 ± 0 | 0 ± 0 |

Values are presented as mean ± SD. Sample sizes per timepoint were as follows: **d1** (Con n=10, LFe n=9, HFe n=10, HCu n=10, HZn n=7), **d6** (Con n=9, LFe n=10, HFe n=10, HCu n=9, HZn n=8), **d12** (Con n=10, LFe n=10, HFe n=8, HCu n=8, HZn n=9), **d15** (Con n=8, LFe n=9, HFe n=10, HCu n=9, HZn n=10), **d18** (Con n=7, LFe n=8, HFe n=9, HCu n=10, HZn n=8), and **d24** (Con n=8, LFe n=8, HFe n=9, HCu n=10, HZn n=9).

Con, Control diet; LFe, Low Iron Diet; HFe, High Iron diet; HCu, High Copper diet; HZn, High Zinc diet

**Table S4.** Relative abundances of genera at d1, 6, 12, 15, 18, and 24

| **Genus** | **Day** | **Con** | **LFe** | **HFe** | **HCu** | **HZn** |
| --- | --- | --- | --- | --- | --- | --- |
| *Prevotella* | 1 | 5.4 ± 4.3 | 6.6 ± 6.3 | 3.4 ± 2.4 | 4 ± 3.8 | 4.8 ± 4.3 |
|  | 6 | 4.5 ± 5.4 | 6.6 ± 5.8 | 5 ± 5.3 | 2.2 ± 3.4 | 20.4 ± 24.1 |
|  | 12 | 12.4 ± 15 | 21.2 ± 17.3 | 10.4 ± 6.2 | 11.6 ± 9.1 | 32.6 ± 14.1 |
|  | 15 | 14.6 ± 12.3 | 23.9 ± 13.2 | 12.4 ± 8.7 | 13 ± 11.5 | 30.9 ± 15.2 |
|  | 18 | 22.6 ± 17.2 | 26.6 ± 17.7 | 13 ± 8.7 | 27.5 ± 21.1 | 24 ± 19.9 |
|  | 24 | 18.2 ± 10.6 | 29.9 ± 17.1 | 20.3 ± 9.3 | 22.6 ± 20.7 | 28.8 ± 15.2 |
| *Bacteroides* | 1 | 11.9 ± 14.4 | 13.6 ± 14.2 | 24.6 ± 16.3 | 17.8 ± 14 | 20.1 ± 21 |
|  | 6 | 11.3 ± 13.7 | 7.6 ± 6.4 | 4.8 ± 3.2 | 5.6 ± 3.7 | 11.5 ± 13.6 |
|  | 12 | 9 ± 12.6 | 3.5 ± 3.9 | 10.8 ± 11.6 | 6.7 ± 5.2 | 18.1 ± 17.9 |
|  | 15 | 7.4 ± 9 | 6.3 ± 9.8 | 11.8 ± 11 | 15.3 ± 20.9 | 12.5 ± 10.7 |
|  | 18 | 7.2 ± 5.3 | 5.9 ± 8.3 | 10.2 ± 8.5 | 6.3 ± 10 | 37.1 ± 26.2 |
|  | 24 | 7.5 ± 7.7 | 2.1 ± 2.7 | 8.8 ± 8.2 | 3.9 ± 4.5 | 32.9 ± 25.6 |
| *Treponema* | 1 | 10.7 ± 14.4 | 8.2 ± 7.1 | 2.7 ± 4.7 | 3.5 ± 4.9 | 5.2 ± 10.8 |
|  | 6 | 18.1 ± 21.4 | 24.1 ± 13.5 | 21.1 ± 13.9 | 28.4 ± 26.6 | 5.6 ± 10.9 |
|  | 12 | 17.3 ± 12.3 | 12.2 ± 12.8 | 16 ± 13.8 | 15.3 ± 10 | 0 ± 0.1 |
|  | 15 | 7 ± 7.8 | 5 ± 6.4 | 4.1 ± 4.1 | 6.1 ± 7.5 | 0 ± 0.1 |
|  | 18 | 3.4 ± 3.1 | 9.6 ± 10.2 | 7.9 ± 8.4 | 8.9 ± 9.1 | 0 ± 0 |
|  | 24 | 1.1 ± 1 | 5.8 ± 10.2 | 4.2 ± 3.3 | 6 ± 9.9 | 0 ± 0 |
| *Prevotellaceae NK3B31 group* | 1 | 2.3 ± 1.4 | 2.6 ± 2.1 | 1.9 ± 1.8 | 3.1 ± 4.3 | 3.2 ± 2.3 |
|  | 6 | 1.6 ± 1.1 | 3.8 ± 2.3 | 2.8 ± 1.7 | 3 ± 2.3 | 7.4 ± 8.8 |
|  | 12 | 3.4 ± 2.7 | 3.9 ± 2.8 | 6.6 ± 2.8 | 6 ± 2.8 | 7.5 ± 8.4 |
|  | 15 | 3.3 ± 2.4 | 5.5 ± 3.1 | 6.3 ± 5.1 | 8.6 ± 6.7 | 16.9 ± 14.3 |
|  | 18 | 8.3 ± 5.6 | 8.9 ± 4.9 | 10.4 ± 8.1 | 8.4 ± 6.4 | 11.8 ± 9.5 |
|  | 24 | 10.6 ± 5.2 | 7.9 ± 5.1 | 11.4 ± 4.9 | 15.5 ± 9.8 | 13.3 ± 14.5 |
| *Ruminococcus* | 1 | 2.9 ± 2.4 | 2.7 ± 2.1 | 2.3 ± 2.3 | 1.9 ± 1.8 | 1.5 ± 1.5 |
|  | 6 | 3.9 ± 3.1 | 5.5 ± 4.9 | 5.8 ± 2.2 | 5 ± 1.4 | 1 ± 1.8 |
|  | 12 | 5.9 ± 3.6 | 5.2 ± 3.5 | 7.4 ± 3.7 | 6.9 ± 4.6 | 0 ± 0 |
|  | 15 | 9.8 ± 6.6 | 5.6 ± 4.8 | 8.9 ± 6.7 | 6.5 ± 4.7 | 0 ± 0.1 |
|  | 18 | 9.1 ± 7.6 | 9.7 ± 3.9 | 9.9 ± 6.2 | 7.6 ± 4.9 | 0 ± 0 |
|  | 24 | 8.8 ± 5.2 | 9.7 ± 5.1 | 9.9 ± 8.4 | 9.8 ± 5.5 | 0.1 ± 0.1 |
| *Alloprevotella* | 1 | 3.7 ± 5.4 | 3 ± 3.4 | 1.9 ± 3.8 | 2 ± 2.3 | 2.1 ± 3.2 |
|  | 6 | 1.8 ± 2.8 | 3.4 ± 3.8 | 2.3 ± 3 | 2.5 ± 3.1 | 4.6 ± 7.2 |
|  | 12 | 3.6 ± 3.2 | 3.8 ± 3.6 | 3.7 ± 2.6 | 4.4 ± 2.9 | 5.3 ± 4.3 |
|  | 15 | 2.3 ± 1.5 | 4.9 ± 5 | 5 ± 5.2 | 6.1 ± 6.1 | 5.9 ± 4.9 |
|  | 18 | 4.2 ± 4 | 3.7 ± 3.2 | 4.5 ± 3.1 | 4.3 ± 2.7 | 10.7 ± 8.7 |
|  | 24 | 5.3 ± 5.6 | 5.3 ± 6.2 | 8.4 ± 7.1 | 8.1 ± 4.9 | 5.3 ± 4.5 |
| *Muribaculaceae* | 1 | 4.1 ± 3.4 | 10 ± 8.7 | 4.8 ± 3.5 | 7 ± 5.9 | 4.4 ± 4.2 |
|  | 6 | 4.2 ± 3.2 | 5.3 ± 4.8 | 5.2 ± 8.2 | 3 ± 2.2 | 3 ± 3.7 |
|  | 12 | 5.7 ± 4 | 2.9 ± 1.5 | 5.8 ± 3.7 | 6.3 ± 6.6 | 6.2 ± 5.3 |
|  | 15 | 9.4 ± 8.2 | 10.2 ± 16.7 | 6.9 ± 6.5 | 8.2 ± 6.7 | 5.9 ± 5.6 |
|  | 18 | 4.2 ± 7.2 | 2.3 ± 5.7 | 1.6 ± 4.6 | 1.2 ± 2.3 | 0.2 ± 0.4 |
|  | 24 | 0 ± 0.1 | 0.1 ± 0.1 | 0 ± 0 | 0 ± 0.1 | 0 ± 0 |
| *Rikenellaceae RC9 gut group* | 1 | 1.5 ± 2.3 | 3.5 ± 5.8 | 2.7 ± 2.5 | 10.1 ± 9.9 | 2.8 ± 2.5 |
|  | 6 | 4 ± 4.1 | 5.7 ± 4.6 | 7.4 ± 8.1 | 4 ± 5.8 | 1.3 ± 2.6 |
|  | 12 | 3.7 ± 3.2 | 3.9 ± 2.9 | 7.6 ± 5.1 | 4.1 ± 1.7 | 0 ± 0.1 |
|  | 15 | 7.6 ± 4.8 | 5.7 ± 3.9 | 6.2 ± 3.1 | 6.6 ± 4.9 | 0.1 ± 0.1 |
|  | 18 | 3.8 ± 3 | 2.7 ± 2.3 | 13.5 ± 16.5 | 3.7 ± 3.3 | 0 ± 0 |
|  | 24 | 5.3 ± 6 | 1.6 ± 1.4 | 5.8 ± 4.2 | 2.7 ± 2.8 | 0 ± 0 |
| *Prevotellaceae UCG-003* | 1 | 0.4 ± 0.5 | 1.5 ± 1.8 | 0.8 ± 1 | 0.5 ± 0.5 | 0.1 ± 0.2 |
|  | 6 | 0.5 ± 0.4 | 2.2 ± 1.7 | 1.9 ± 3.4 | 0.4 ± 0.4 | 0.9 ± 1.2 |
|  | 12 | 2.2 ± 2.5 | 3.7 ± 2.7 | 3.6 ± 4.7 | 3.8 ± 4.1 | 7.2 ± 8.2 |
|  | 15 | 4.5 ± 5.6 | 5.3 ± 4.7 | 4.4 ± 6.3 | 4.1 ± 4.4 | 3.4 ± 4.2 |
|  | 18 | 5.6 ± 5.1 | 8.1 ± 8.5 | 5 ± 3.7 | 7.7 ± 9.9 | 1 ± 2 |
|  | 24 | 6 ± 4.7 | 5.4 ± 6.9 | 5 ± 3.6 | 5.7 ± 4.1 | 1.4 ± 3.1 |
| *Escherichia-Shigella* | 1 | 7.5 ± 11.1 | 4.2 ± 7 | 4.8 ± 4.9 | 5.4 ± 6.9 | 6.4 ± 4.9 |
|  | 6 | 1.3 ± 2 | 1.6 ± 1.9 | 3.8 ± 6.8 | 6.6 ± 11.2 | 8.8 ± 12.6 |
|  | 12 | 4 ± 7.9 | 11.7 ± 21.7 | 1 ± 1.4 | 3.2 ± 3.1 | 0.5 ± 0.8 |
|  | 15 | 1.3 ± 1.4 | 2.2 ± 2.9 | 3 ± 7.3 | 1.9 ± 2 | 2.9 ± 3.5 |
|  | 18 | 0.9 ± 1.3 | 0.8 ± 0.7 | 0.5 ± 0.6 | 4.1 ± 8 | 0.5 ± 0.9 |
|  | 24 | 0.7 ± 1.2 | 0.7 ± 0.9 | 1.3 ± 2.2 | 1.5 ± 3.5 | 0.2 ± 0.4 |
| *Succinivibrio* | 1 | 2.5 ± 4.2 | 3 ± 3.1 | 1.4 ± 2.7 | 0.4 ± 0.9 | 2.7 ± 5.1 |
|  | 6 | 10.1 ± 12.4 | 1.7 ± 1.7 | 2.3 ± 3.6 | 4.8 ± 5.5 | 1 ± 2.6 |
|  | 12 | 2.1 ± 2.5 | 4.6 ± 5.7 | 4 ± 3.6 | 6.2 ± 9.9 | 0.9 ± 2.7 |
|  | 15 | 1.9 ± 4.3 | 4.1 ± 6.6 | 2.9 ± 3.7 | 4.1 ± 5.9 | 0.1 ± 0.2 |
|  | 18 | 1.4 ± 2.4 | 1.7 ± 2.5 | 1.2 ± 1.9 | 3.4 ± 4.4 | 0.1 ± 0.2 |
|  | 24 | 3.1 ± 4.6 | 5.5 ± 8.4 | 0.5 ± 0.9 | 3.5 ± 5.6 | 0 ± 0.1 |
| *Parabacteroides* | 1 | 1.4 ± 2.7 | 0.8 ± 1.1 | 4.9 ± 7 | 2.5 ± 2.9 | 2 ± 2.6 |
|  | 6 | 4.3 ± 4 | 2.2 ± 3.8 | 5.3 ± 8.3 | 2.8 ± 3.4 | 2.3 ± 2.2 |
|  | 12 | 0.5 ± 0.8 | 0.9 ± 1.1 | 1.1 ± 1.7 | 0.4 ± 0.6 | 3.3 ± 2.5 |
|  | 15 | 0.8 ± 1 | 1.5 ± 1 | 1.4 ± 2.3 | 1.8 ± 3.8 | 7.5 ± 11.8 |
|  | 18 | 1.6 ± 1.9 | 1.1 ± 1 | 1.6 ± 1.5 | 0.7 ± 1 | 5.6 ± 3.9 |
|  | 24 | 2.7 ± 2 | 1.4 ± 1.6 | 2.8 ± 3.4 | 1.1 ± 0.9 | 3.4 ± 3.9 |
| *Christensenellaceae R-7 group* | 1 | 6.8 ± 9.4 | 4.7 ± 7 | 10.6 ± 14.8 | 5.8 ± 5.8 | 6.1 ± 6 |
|  | 6 | 2.5 ± 3.7 | 3.3 ± 5.9 | 4 ± 4.6 | 3 ± 5.2 | 2.4 ± 6.3 |
|  | 12 | 1.6 ± 4.1 | 0.3 ± 0.6 | 0.1 ± 0.2 | 0.1 ± 0.2 | 0 ± 0 |
|  | 15 | 4.4 ± 5.5 | 0.2 ± 0.2 | 1.7 ± 5.1 | 0.2 ± 0.5 | 0 ± 0 |
|  | 18 | 2.7 ± 5.7 | 0.8 ± 1.3 | 0.2 ± 0.3 | 0.3 ± 0.7 | 0 ± 0 |
|  | 24 | 4.3 ± 11.7 | 2.7 ± 6.8 | 0 ± 0 | 0.1 ± 0.2 | 0 ± 0 |
| *Clostridium sensu stricto 1* | 1 | 1.1 ± 1.5 | 1.4 ± 1.4 | 0.3 ± 0.4 | 1 ± 1.4 | 0.8 ± 1.3 |
|  | 6 | 0.8 ± 1.4 | 0.4 ± 0.4 | 1.3 ± 2.5 | 0.3 ± 0.4 | 0.2 ± 0.3 |
|  | 12 | 0.3 ± 0.4 | 0.2 ± 0.2 | 1.2 ± 1.1 | 1.9 ± 4 | 1.8 ± 2.1 |
|  | 15 | 0.5 ± 1 | 1.6 ± 2.7 | 1.6 ± 2.1 | 0.3 ± 0.6 | 2.9 ± 2.8 |
|  | 18 | 1.9 ± 2 | 2.8 ± 4.3 | 1.9 ± 1.9 | 0.1 ± 0.1 | 1.8 ± 2.3 |
|  | 24 | 0.8 ± 0.7 | 1.1 ± 1.2 | 1.7 ± 1.7 | 1 ± 1.2 | 6.3 ± 6.6 |
| *Clostridia vadinBB60 group* | 1 | 1.3 ± 1.5 | 2.5 ± 3.5 | 2.1 ± 2.3 | 0.8 ± 1 | 1.7 ± 3.9 |
|  | 6 | 0.7 ± 0.9 | 1.5 ± 1.6 | 1.5 ± 2.1 | 0.9 ± 0.7 | 0.5 ± 1 |
|  | 12 | 2 ± 2.9 | 2.4 ± 3.3 | 0.6 ± 0.8 | 1.1 ± 1.6 | 0.2 ± 0.4 |
|  | 15 | 2.4 ± 2.4 | 2.6 ± 4.2 | 4 ± 8.4 | 1 ± 1.5 | 0.4 ± 0.8 |
|  | 18 | 0.9 ± 0.9 | 0.9 ± 1 | 1.5 ± 1.4 | 0.1 ± 0.2 | 0.5 ± 1.1 |
|  | 24 | 0.8 ± 0.9 | 0.6 ± 0.8 | 0.6 ± 0.5 | 0.2 ± 0.3 | 0.1 ± 0.1 |
| *[Eubacterium] coprostanoligenes group* | 1 | 2.7 ± 5.7 | 1.4 ± 1.5 | 2.1 ± 3.3 | 2.7 ± 5.2 | 0.7 ± 0.6 |
|  | 6 | 1.5 ± 2.7 | 0.3 ± 0.3 | 0.5 ± 0.5 | 1.4 ± 1.6 | 0.6 ± 1 |
|  | 12 | 0.7 ± 0.7 | 1 ± 1.3 | 1.3 ± 0.9 | 0.8 ± 1 | 0.8 ± 0.9 |
|  | 15 | 1.7 ± 1.3 | 1.1 ± 1.2 | 1.1 ± 0.7 | 0.8 ± 0.6 | 0.6 ± 1 |
|  | 18 | 1.4 ± 1.2 | 1.2 ± 1 | 1 ± 0.6 | 1.2 ± 0.6 | 0.3 ± 0.6 |
|  | 24 | 0.8 ± 1.1 | 1.7 ± 1.6 | 1.5 ± 0.6 | 1.8 ± 1.9 | 0.8 ± 1 |
| *dgA-11 gut group* | 1 | 0.1 ± 0.2 | 0.8 ± 0.9 | 0.4 ± 0.6 | 1 ± 1.8 | 0.1 ± 0.2 |
|  | 6 | 0.9 ± 1.5 | 0.9 ± 1.1 | 0.8 ± 1.5 | 0.5 ± 0.6 | 0.2 ± 0.7 |
|  | 12 | 1.7 ± 2.4 | 1.3 ± 1.3 | 1.5 ± 1.6 | 3.3 ± 6.9 | 0 ± 0 |
|  | 15 | 1.8 ± 1.2 | 1.5 ± 1.5 | 2 ± 1.9 | 0.8 ± 1.1 | 0 ± 0 |
|  | 18 | 1.7 ± 1.9 | 2.5 ± 2.6 | 2.7 ± 2.2 | 1 ± 1.3 | 0 ± 0 |
|  | 24 | 1 ± 1.3 | 1.1 ± 1.8 | 2 ± 2 | 1.7 ± 3.1 | 0 ± 0 |
| *Lactobacillus* | 1 | 3.5 ± 6.8 | 2 ± 4.6 | 0.6 ± 1.3 | 3.4 ± 7.2 | 1.2 ± 2.2 |
|  | 6 | 4.5 ± 7.4 | 2.5 ± 3 | 4.1 ± 8 | 0 ± 0 | 5.1 ± 4.7 |
|  | 12 | 0.9 ± 1.8 | 1.8 ± 3.7 | 0.2 ± 0.4 | 0.1 ± 0.3 | 1.8 ± 4.6 |
|  | 15 | 0.1 ± 0.1 | 0 ± 0 | 0 ± 0.1 | 0 ± 0 | 0.1 ± 0.2 |
|  | 18 | 0 ± 0 | 0 ± 0 | 0 ± 0 | 0 ± 0 | 0 ± 0 |
|  | 24 | 0 ± 0 | 0 ± 0 | 0 ± 0 | 0 ± 0 | 0 ± 0 |

Values are presented as mean ± SD.

Con, Control diet; LFe, Low Iron Diet; HFe, High Iron diet; HCu, High Copper diet; HZn, High Zinc diet

**
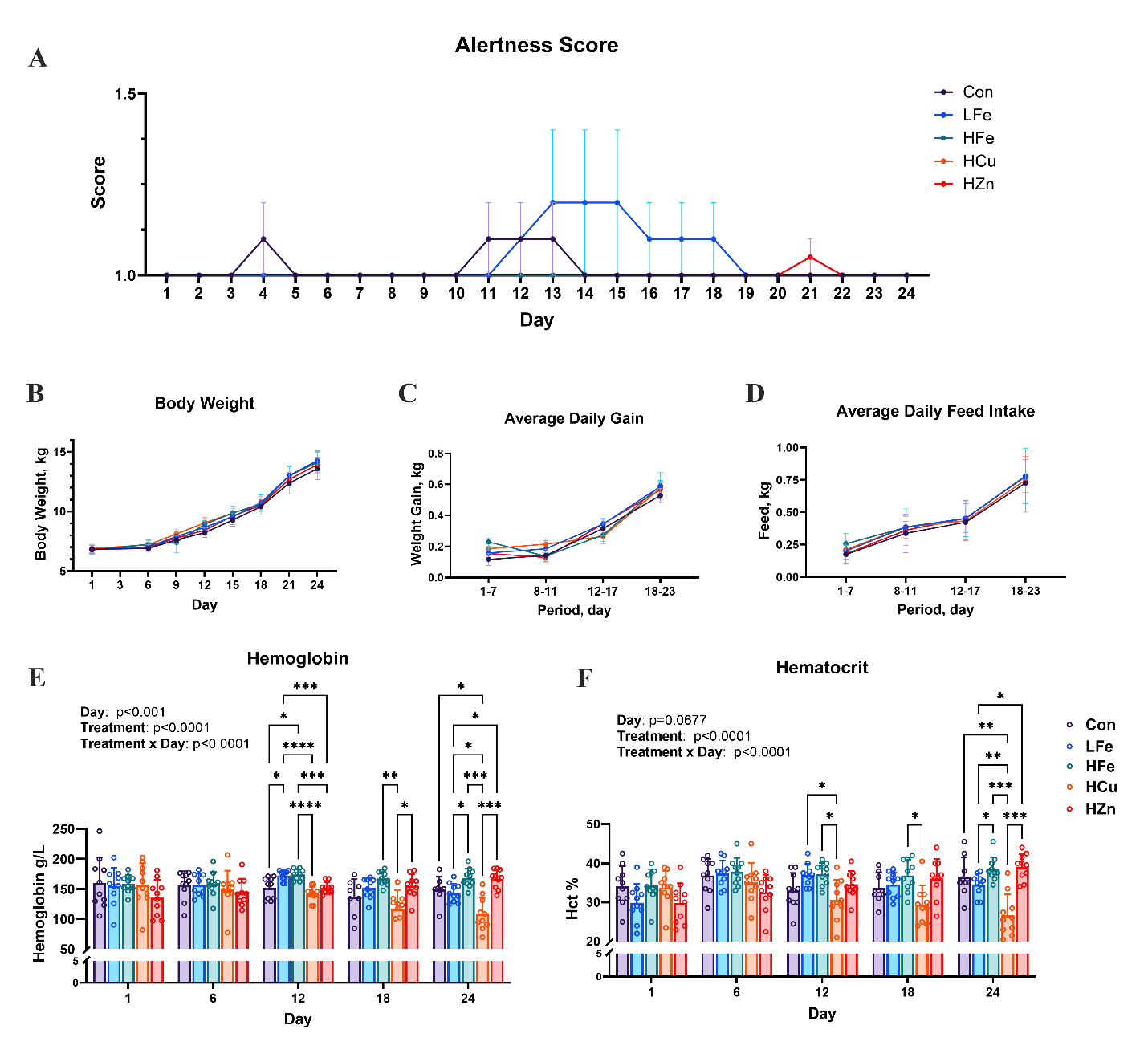
**

**Figure S1.** Effects of dietary treatments on growth performance, feed efficiency, and hematological parameters. **(A)** Alertness score assessed on a 3-point scale, **(B)** Body weight (kg), **(C)** Average daily gain (ADG, kg). **(D)** Average daily feed intake (ADFI, kg), **(E)** Hemoglobin (g/L) on days 1, 6, 12, 18, 24. (F) Hematocrit (%) on days 1, 6, 12, 18, 24. ADG and ADFI were assessed across four periods: days 1-7, 8-11, 12-17, and 18-23. Pairwise comparison: *, p< 0.05; **, p< 0.01; ***, p<0.001; ****, p< 0.0001.

Con, Control diet; LFe, Low Iron Diet; HFe, High Iron diet; HCu, High Copper diet; HZn, High Zinc diet
